# Marcus kinetics control singlet and triplet oxygen evolving from superoxide

**DOI:** 10.1038/s41586-025-09587-7

**Published:** 2025-10-03

**Authors:** Soumyadip Mondal, Huyen T. K. Nguyen, Robert Hauschild, Stefan A. Freunberger

**Affiliations:** 1 Institute of Science and Technology Austria (ISTA), Klosterneuburg, Austria; 2 Present Address: Texas Materials Institute, The University of Texas at Austin, Austin TX, USA

**Keywords:** Energy, Electrochemistry, Electron transfer, Reaction kinetics and dynamics

## Abstract

Oxygen redox chemistry is central to life^1^ and many human-made technologies, such as in energy storage^2–4^. The large energy gain from oxygen redox reactions is often connected with the occurrence of harmful reactive oxygen species^3,5,6^. Key species are superoxide and the highly reactive singlet oxygen^3–7^, which may evolve from superoxide. However, the factors determining the formation of singlet oxygen, rather than the relatively unreactive triplet oxygen, are unknown. Here we report that the release of triplet or singlet oxygen is governed by individual Marcus normal and inverted region behaviour. We found that as the driving force for the reaction increases, the initially dominant evolution of triplet oxygen slows down, and singlet oxygen evolution becomes predominant with higher maximum kinetics. This behaviour also applies to the widely observed superoxide disproportionation, in which one superoxide is oxidized by another, in both non-aqueous and aqueous systems, with Lewis and Brønsted acidity controlling the driving forces. Singlet oxygen yields governed by these conditions are relevant, for example, in batteries or cellular organelles in which superoxide forms. Our findings suggest ways to understand and control spin states and kinetics in oxygen redox chemistry, with implications for fields, including life sciences, pure chemistry and energy storage.

## Abstract (web summary)

The release of triplet or singlet oxygen is governed by individual Marcus normal and inverted region behaviour.

## Main

More than half a century after the discovery that singlet oxygen forms from superoxide^8^, what governs the evolution of singlet or triplet oxygen under many conditions relevant to life and human-made oxygen redox systems remains unknown. Oxygen redox chemistry is crucial to life^1^ and encompasses some of the most fundamental and widespread chemical reactions, including those in batteries^2–4,9^, fuel cells and electrocatalysis^10^, and organic chemistry^11^. The oxidation states that are accessed in these reactions range from 0 (dioxygen) to –2 (oxide), with intermediate redox states of –½ and –1, pertaining to superoxide (O_2_^−^) and peroxide (O_2_^2−^) or the oxide radical (O^•–^). Superoxide is pivotal in oxygen reduction and evolution reactions because it is the closest oxidation state to dioxygen. Dioxygen appears either in its ground state as triplet oxygen (^3^O_2_ or ^3^Σ_g_^–^) or as singlet oxygen (^1^O_2_ or ^1^Δ_g_) in its first electronically excited state. Whereas ^3^O_2_ is relatively unreactive, ^1^O_2_ is highly reactive with most organic matter^11,12^. This is readily evidenced in metal-ion batteries^6,7^ and metal-O_2_ batteries^3,4^, in which ^1^O_2_ is the primary source of degradation, causing decomposition of organic electrolytes and conductive carbon additives, which ultimately degrades the overall device function. Moreover, ^1^O_2_ and (su)peroxide are well-known reactive oxygen species in biological systems and are involved in several processes from signalling to cell damage^1,5^.

Superoxide liberates oxygen under a broad range of oxidizing conditions. A prevalent process is disproportionation due to the instability of superoxides in most of the environments^3,9,13^. This reaction occurs in both protic (aqueous)^14,15^ and aprotic environments with relatively strong Lewis acids such as Li^+^ and Na^+^ (refs. ^6,9,13,16^). During disproportionation (2O_2_^−^ → O_2_ + O_2_^2−^), one superoxide molecule is reduced to peroxide, whereas the other is oxidized to form either ^3^O_2_ or ^1^O_2_. Examples can be found in both cellular respiration^1,5^ and batteries^13,16–20^. Relative ^3^O_2_ and ^1^O_2_ yields and kinetics of superoxide disproportionation, and superoxide oxidation more generally, are therefore fundamental to these systems, but underlying mechanisms are still unknown.

Here we examined ^1^O_2_ evolution from the oxidation of superoxide through both chemical reaction and disproportionation over a wide range of driving forces. On the basis of this, we observe distinct Marcus normal and inverted region behaviour for ^3^O_2_ and ^1^O_2_ evolution, which explains ^1^O_2_ formation over a broad range of scenarios in which superoxide oxidation occurs, for example, in biological systems and energy storage.

## Excited species through electron transfer

Superoxide oxidation may form both ^3^O_2_ and ^1^O_2_ according to Wigner–Witmer spin conservation rules^21^. This applies to disproportionation, in which two doublet superoxides can produce triplet or singlet products^17^, and to oxidation by a redox mediator (RM^ox^) to form one excited (^1^O_2_) and one ground state molecule (the reduced mediator, RM^red^) (ref. ^22^).

**Extended Data Fig. 1:**
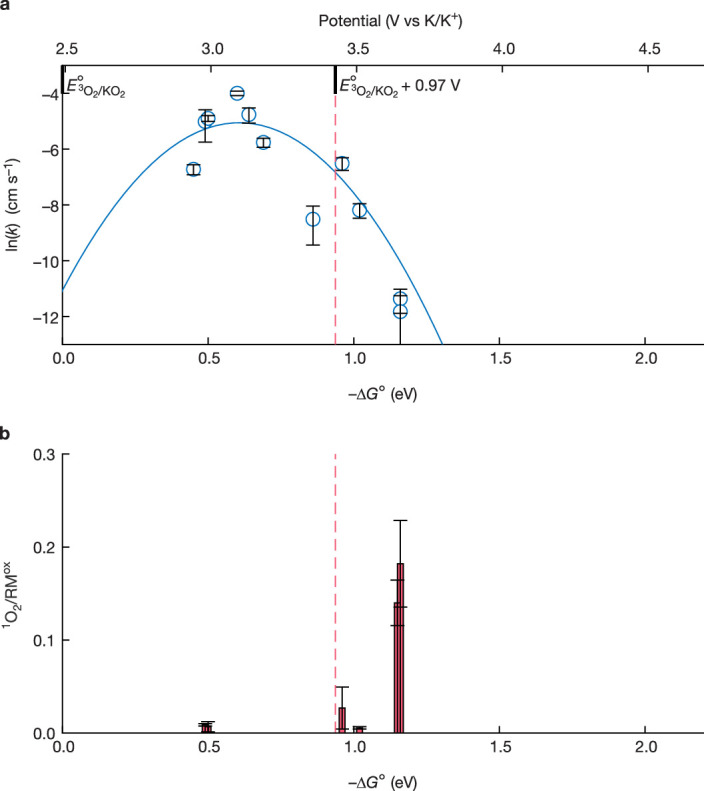
Kinetics and ^1^O_2_ yields during mediated superoxide oxidation in TEGDME electrolyte. **a**, We measured the kinetic constants *k* for mediated KO_2_ oxidation with mediators using UV-Vis spectroscopy up to moderate mediator redox potentials. Higher potential mediators could not be used in the tetraglyme electrolyte, and errors were already significant at the upper end of driving forces. Plot of ln(*k*) versus mediator potential (${E}_{{\text{RM}}^{\text{ox}/\text{red}}}^{\circ }$, top axis) and driving force ($-\Delta G^\circ$, bottom axis). $-\Delta G^\circ =({E}_{{\text{RM}}^{\text{ox}/\text{red}}}^{\circ }-{E}_{{{}^{3}{\rm{O}}}_{2}/{\text{KO}}_{2}}^{\circ })F$, where ${E}_{{{}^{3}{\rm{O}}}_{2}/{\text{KO}}_{2}}^{\circ }=2.48\,{\rm{V}}$ vs K/K^+^. The blue curve is a fit to Marcus kinetics in Eq. (1), which gives *Z*_el_ = 6.4 × 10^–3^ cm s^–1^ and $\lambda$ = 0.61 eV. The red dashed line indicates the driving force at ${E}_{{{}^{1}{\rm{O}}}_{2}/{\text{KO}}_{2}}^{\circ }={E}_{{{}^{3}{\rm{O}}}_{2}/{\text{KO}}_{2}}^{\circ }+0.97{\rm{V}}$, the commonly expected “threshold” for ^1^O_2_ formation. **b**, The formed ^1^O_2_ per mole of RM^ox^ as measured using 9,10-dimethylanthracene (DMA) conversion to DMA-O_2_ using HPLC. Data are presented as mean ± s.d. (*n* ≥ 3). Panel **a** and **b** adapted from ref. ^18^, Springer Nature Limited.

We previously investigated the mediated oxidation of superoxide through the reaction KO_2_ + RM^ox^ → O_2_ + RM^red^ + K^+^ in the search for possible ^1^O_2_ evolution^18^. The potential ${E}_{{\text{RM}}^{\text{ox}/\text{red}}}^{\circ }$ was limited to moderate values because of the stability of the ether electrolyte used. We measured ^3^O_2_ and ^1^O_2_ yields using mass spectrometry and a chemical trap, respectively, and tracked the reaction kinetics using ultraviolet–visible (UV–Vis) spectroscopy. The data shown in Extended Data Fig. 1 supported two main conclusions: first, ^1^O_2_ was observed with RMs with ${E}_{{\text{RM}}^{\text{ox}/\text{red}}}^{\circ }\gtrsim {E}_{{\genfrac{}{}{0ex}{}{3}{}{\rm{O}}}_{2}/{\text{KO}}_{2}}^{\circ }+1\,{\rm{V}}$, which could be in accordance with the frequently quoted threshold^3,23–26^ for ^1^O_2_ evolution based on the energy difference of 0.97 eV between ^3^O_2_ and ^1^O_2_; and second, the kinetics of superoxide oxidation over the measured range of driving forces exhibited Marcus normal and inverted region behaviour. This behaviour is characterized by a parabolic relationship between the logarithm of the kinetic constant *k* and the driving force (free energy change) $-\Delta {G}^{\circ }=\left({E}_{{\text{RM}}^{\text{ox}/\text{red}}}^{\circ }-{E}_{{\genfrac{}{}{0ex}{}{3}{}{\rm{O}}}_{2}/{\text{KO}}_{2}}^{\circ }\right)F$:

1 $$k={Z}_{{\rm{el}}}\cdot {{\rm{e}}}^{\frac{-(\Delta {G}^{\circ }+\lambda {)}^{2}}{4RT\lambda }}$$

where *Z*_el_ is the collision factor, *λ* is the reorganization energy and *F* is the Faraday constant^22,27,28^. The kinetic constant *k* reaches a peak when –Δ*G*° = *λ* and electron transfer becomes barrierless. The inverted region results from growing barriers at even higher driving forces. The data suggested that superoxide oxidation kinetics follow a single parabola and that some ^1^O_2_ is generated for –Δ*G*° ≳ 0.97 eV. However, what controls the extent to which ^1^O_2_ or ^3^O_2_ evolve is yet unknown.

**Fig. 1:**
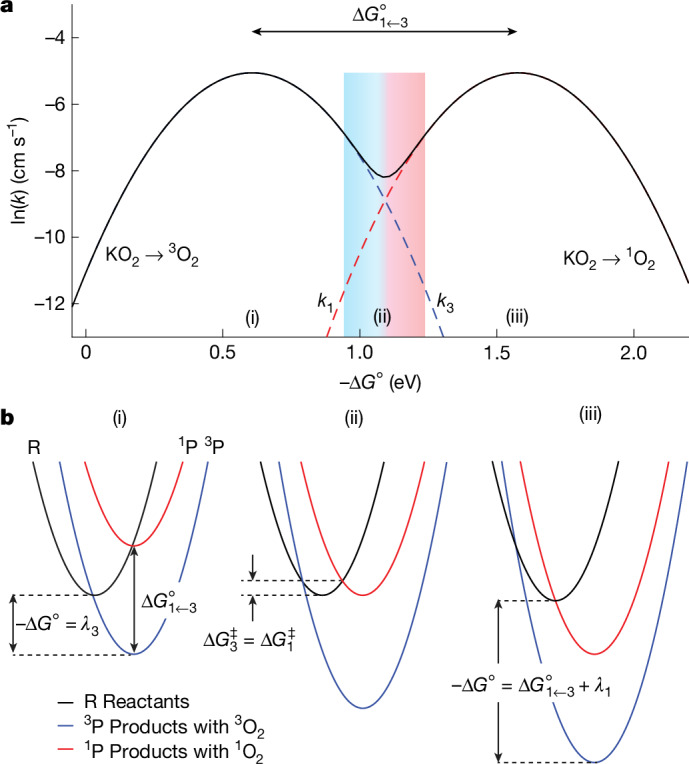
Marcus theory suggests separate kinetics of superoxide oxidation to ^3^O_2_ and ^1^O_2_. **a**, Hypothesis for how the driving force could govern ^3^O_2_ and ^1^O_2_ formation kinetics from superoxide oxidation. The left parabola results from previously measured rate constants for mediated superoxide oxidation with driving forces up to –Δ*G*° ≈ 1.2 eV as shown in Extended Data Fig. 1. However, a single kinetic parabola could not conclusively explain why ^1^O_2_ formed for –Δ*G*° ≳ 1 eV. Based on the considerations in **b**, individual kinetic parabolas (*k*_3_ and *k*_1_) for the reactions yielding ^3^O_2_ and ^1^O_2_ can be constructed with the full line showing their sum. The maxima are shifted by ${\Delta G}_{1\leftarrow 3}^{\circ }\approx 0.97{\rm{eV}}$ (see text), and equal prefactors and reorganization energies are assumed. The blue- and red-shaded area shows the transition from *k*_3_/(*k*_1+3_) = 0.99 to *k*_1_/(*k*_1+3_) = 0.99. **b**, Potential energy surfaces for mediated superoxide oxidation for different driving forces. Black, blue and red parabolas denote reactants (KO_2_ + RM^ox^) and ^3^O_2_ or ^1^O_2_ in the products (^3^O_2_ + RM^red^ or ^1^O_2_ + RM^red^), respectively. The cases shown are for barrierless reactions to ^3^O_2_ (i) and ^1^O_2_ (iii) and equal barriers (ii). The subscripts 3 and 1 denote triplet and singlet states, respectively. Source Data

^1^O_2_ evolution should be a distinct elementary step both thermodynamically and kinetically. We, therefore, propose that the observed parabola in Extended Data Fig. 1 describes oxidation to ^3^O_2_, and a second parabola should appear at sufficiently large driving forces, representing oxidation to ^1^O_2_ (Fig. 1a). The intersection of these two parabolas would indicate the transition from ^3^O_2_ to ^1^O_2_. This follows classical work by Marcus on the electrogeneration of electronically excited species^22,27^. However, this hypothesis has not yet been experimentally evaluated for ^1^O_2_ generation.

Based on equation (1), a kinetic expression with separate terms for ^3^O_2_ and ^1^O_2_ evolution can be given as

2 $${k}_{1+3}={k}_{3}+{k}_{1}={Z}_{{\rm{el}},3}\cdot {{\rm{e}}}^{\frac{-{(\Delta {G}^{\circ }+{\lambda }_{3})}^{2}}{4RT{\lambda }_{3}}}+{Z}_{{\rm{el}},1}\cdot {{\rm{e}}}^{\frac{-{(\Delta {G}^{\circ }+{\Delta G}_{1\leftarrow 3}^{\circ }+{\lambda }_{1})}^{2}}{4RT{\lambda }_{1}}}.$$

The subscripts 3 and 1 on *k*, *Z*_el_ and *λ* denote values for ^3^O_2_ and ^1^O_2_, respectively. The blue- and red-dashed parabolas in Fig. 1a represent the terms for ^3^O_2_ and ^1^O_2_, whereas the solid line shows their sum. The *k*_3_ parabola results from the transition from the black to the blue potential energy surfaces in Fig. 1b, which are shown for –Δ*G*° ≈ *λ*_3_ for case (i). The vibrational ground states of ^3^O_2_ and ^1^O_2_ differ by ${\Delta H}_{1\leftarrow 3}^{\circ }=\Delta {H}^{\circ }\left(\genfrac{}{}{0ex}{}{1}{}\,{\Delta }_{\text{g}}\leftarrow \genfrac{}{}{0ex}{}{3}{}\,{\Sigma }_{\text{g}}^{-}\right)=0.97\,{\rm{eV}}$, where Δ*H* is the enthalpy change^12^. Assuming, for now, a vanishing entropy change Δ*S* as typically done in electrochemiluminescence literature^28^, ${\Delta G}_{1\leftarrow 3}^{\circ }=\Delta {G}^{\circ }\left(\genfrac{}{}{0ex}{}{1}{}\,{\Delta }_{\text{g}}\leftarrow \genfrac{}{}{0ex}{}{3}{}\,{\Sigma }_{\text{g}}^{-}\right)\approx {\Delta H}_{1\leftarrow 3}^{\circ }$, by which the singlet potential energy surface shifts vertically (Fig. 1b, red). For sufficiently large driving forces, the barriers Δ*G*^‡^ and kinetics for crossing to ^3^O_2_ and ^1^O_2_ become equal (Fig. 1, case (ii)) or even barrierless to ^1^O_2_ (case (iii)). Hence, individual kinetic parabolas for superoxide oxidation to ^3^O_2_ and ^1^O_2_ can be constructed. The hypothetical parabola for ^1^O_2_ evolution can be drawn if we further assume, for now, equal collision factors *Z*_el,1_ = *Z*_el,1_ and equal reorganization energies *λ*_1_ = *λ*_3_ as fitted to the data in Extended Data Fig. 1. The theory developed initially for homogeneous electron transfer can still be used for semiconductors or insulators, as noted by Marcus^22^. Therefore, the considerations in Fig. 1 apply to the oxidation of solid superoxides and superoxide solutions.

## Kinetics over extended driving forces

**Fig. 2:**
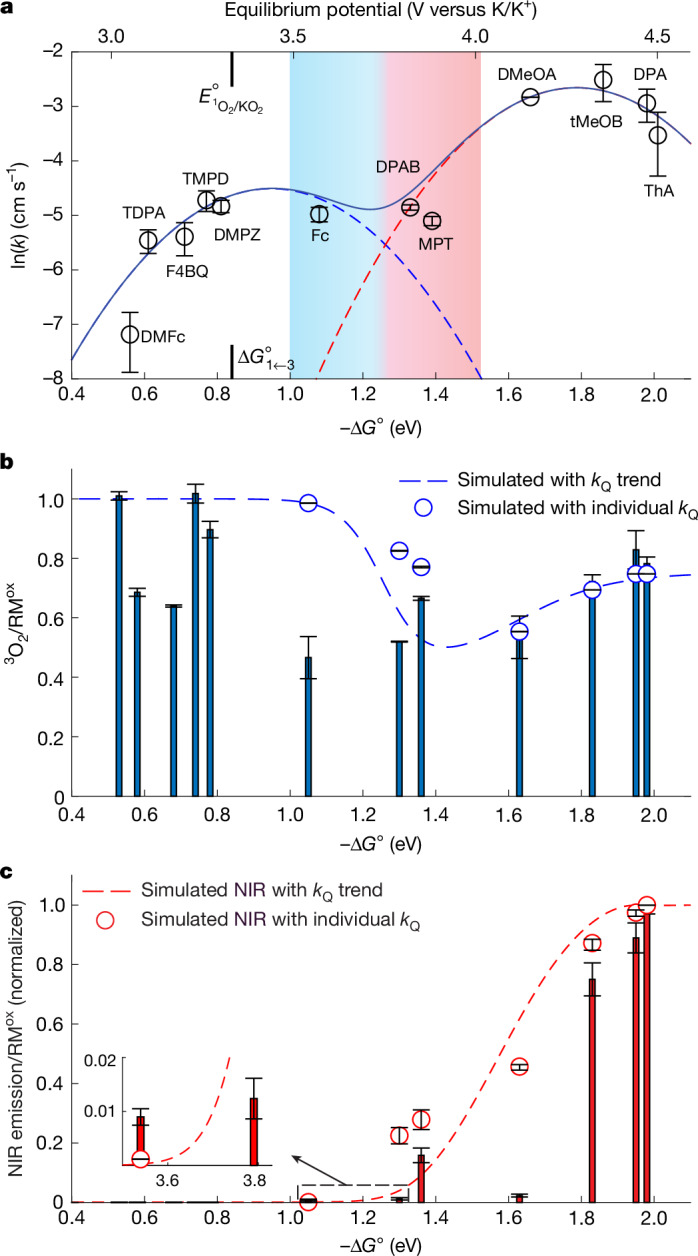
Free energy dependence of superoxide oxidation kinetics to ^3^O_2_ and ^1^O_2_ and their yields. **a**, We measured the kinetic constants *k* for mediated KO_2_ oxidation in MeCN electrolyte with mediators covering a large range of redox potentials. Plot of ln(*k*) compared with the mediator potential (${E}_{{\text{RM}}^{\text{ox}/\text{red}}}^{\circ }$, top axis) and driving force (–Δ*G*°, bottom axis). $-\Delta {G}^{\circ }=\left({E}_{{\text{RM}}^{\text{ox}/\text{red}}}^{\circ }-{E}_{{\genfrac{}{}{0ex}{}{3}{}{\rm{O}}}_{2}/{\text{KO}}_{2}}^{\circ }\right)\,F$, where ${E}_{{\genfrac{}{}{0ex}{}{3}{}{\rm{O}}}_{2}/{\text{KO}}_{2}}^{\circ }=2.48\,{\rm{V}}$ on the K/K^+^ scale. The mediators are shown in Extended Data Fig. 2. The full line best fits equation (2); the broken line parabolas represent the first and second terms in equation (2). The fitted values are *Z*_el,3_ = 1.10 × 10^–2^ cm s^–1^, *Z*_el,1_ = 7.00 × 10^–2^ cm s^–1^, *λ*_3_ = *λ*_1_ = 0.95 eV, ${\Delta G}_{1\leftarrow 3}^{\circ }=0.84\,{\rm{eV}}$ and *R*^2^ = 0.998. Based on these fits, the standard potential ${E}_{{\genfrac{}{}{0ex}{}{1}{}{\rm{O}}}_{2}/{\text{KO}}_{2}}^{\circ }$ and associated driving force ${\Delta G}_{1\leftarrow 3}^{\circ }$ are marked. They are linked by ${E}_{{\genfrac{}{}{0ex}{}{1}{}{\rm{O}}}_{2}/{\text{KO}}_{2}}^{\circ }\,=\,{E}_{{\genfrac{}{}{0ex}{}{3}{}{\rm{O}}}_{2}/{\text{KO}}_{2}}^{\circ }+{\Delta G}_{1\leftarrow 3}^{\circ }/F$. The blue- and red-shaded area indicates the transition from *k*_3_/(*k*_1+3_) = 0.99 to *k*_1_/(*k*_1+3_) = 0.99. **b**, ^3^O_2_ yield per mole of RM^ox^ (bars) during KO_2_ oxidation as measured by mass spectrometry. The dashed line and the circular markers show simulated ^3^O_2_ yields considering ^1^O_2_ quenching by solvent and redox mediator (Methods and Extended Data Fig. 3). The dashed line used the trend line for the mediator quenching rate constant *k*_Q_, whereas the markers use the individually measured values (Extended Data Fig. 3c). **c**, Normalized 1,270 nm NIR emission (bars) during KO_2_ oxidation. The dashed line and the circular markers show the simulated NIR emission considering ^1^O_2_ formation with the kinetics *k*_1_ (the right parabola in **a**) and ^1^O_2_ quenching by solvent and redox mediator (Methods and Extended Data Fig. 3). Data are presented as mean ± s.d. (*n* ≥ 3). Source Data

**Extended Data Fig. 2:**
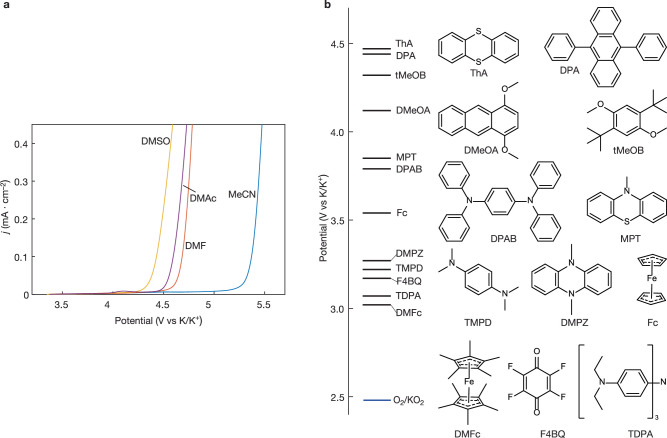
Oxidation stability of different solvents and potentials of the redox mediators used here. **a**, Forward scan of cyclic voltammetry of dimethyl sulfoxide (DMSO), N,N-dimethylacetamide (DMAc), N,N-dimethylformamide (DMF), and acetonitrile (MeCN) containing 10 mM KClO_4_ The scan rate was 20 mVs^–1^ and the working electrode was a glassy carbon disc. **b**, The selected RMs, their abbreviations and measured redox potentials on the K/K^+^ scale as well as *E*° of the O_2_/KO_2_ couple.

To test the hypothesis of individual kinetic parabolas for ^3^O_2_ and ^1^O_2_ evolution, we selected acetonitrile (MeCN) as the solvent. Acetonitrile is highly stable against oxidation and, therefore, allows for large driving forces (Extended Data Fig. 2a) to be tested. We used a wide range of mediators (Extended Data Fig. 2b) with redox potentials that exceed the expected maximum of the ^1^O_2_ parabola. Figure 2a shows the measured kinetics as a function of driving force relative to ${E}_{{\genfrac{}{}{0ex}{}{3}{}{\rm{O}}}_{2}/{\text{KO}}_{2}}^{\circ }$ (Methods). The measured kinetics can be adequately fitted to equation (2). The blue- and red-dashed parabolas represent the terms for ^3^O_2_ and ^1^O_2_ evolution, whereas the solid line shows their sum. Evidence for these parabolas corresponding to ^3^O_2_ and ^1^O_2_ is provided by measured yields (Fig. 2b,c). The ^3^O_2_ yields were measured by mass spectrometry and quantified as the molar ratio ^3^O_2_/RM^ox^. ^1^O_2_ formation was measured by its specific 1,270 nm near-infrared (NIR) radiation (Methods). Absolute quantification of ^1^O_2_ is difficult, and values were, therefore, normalized to the maximum observed.

**Extended Data Fig. 3:**
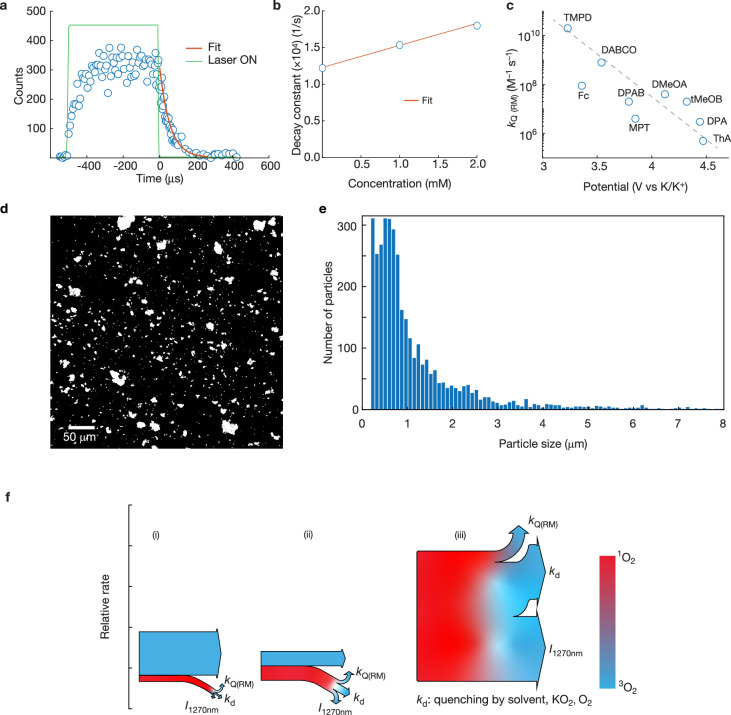
From ^1^O_2_ formation rates to measurable NIR emission intensities. **a**, Example for one mediator of the decay of the ^1^O_2_ phosphorescence at 1270 nm in MeCN after pulsed irradiation. The pulse frequency was 1 kHz and the signal was accumulated for 2 min. **b**, Example for one RM of the decay constant of ^1^O_2_ versus RM^red^ concentration to get *k*_Q(RM)._ Every value at any concentration is the mean of *n* ≥ 3 decay measurements. Error bars are too small to be visible. **c**, Measured ^1^O_2_ quenching rate constants *k*_Q(RM)_ of the used mediators in their reduced form RM^red^ as a function of their redox potential. The trend line with a slope of 10^3.5^/V has been added to guide the eye. Each marker represents one set of measurements as shown in **b**. **d**, Segmented optical microscopy image of dispersed KO_2_ powder. **e**, Extracted particle size distribution from **d**, from which a surface area of *A* = 0.23 ± 0.04 m^2^g^–1^ (mean ± s.d., *n* = 3) was determined. **f**, Schematic Sankey plots of relative rates of the involved processes for the examples of Fc, MPT and ThA. The widths of the arrows are proportional to the rates. Relative rates at the left end are those given by the Marcus parabola for superoxide oxidation to ^3^O_2_ and ^1^O_2_ in Fig. 2a. Any ^1^O_2_ then undergoes various decay pathways. First, a combination of physical and reactive quenching by the solvent, ^3^O_2_ and superoxide, as denoted as *k*_d_. Second, strongly potential-dependent charge transfer quenching by the RM denoted as *k*_q(RM)_. The fraction emitting NIR radiation is strongly exacerbated as it would otherwise be invisible relative to the others. Data are presented in **b** and **c** are from single experiment.

Observable yields of ^3^O_2_/RM^ox^ and NIR intensities do not simply resemble the relative kinetics of ^3^O_2_ and ^1^O_2_ formation as obtained in Fig. 2a. Instead, the formed ^1^O_2_ undergoes multiple decay pathways, of which some yield ^3^O_2_ and only a small fraction emits the NIR radiation^12^. To rationalize the measured values, we simulated them based on formation rates and ^1^O_2_ decay processes (Methods and Extended Data Fig. 3): (1) ^3^O_2_ and ^1^O_2_ formation rates as given by the parabolas in Fig. 2a; (2) physical and reactive ^1^O_2_ quenching by the solvent; (3) physical and reactive ^1^O_2_ quenching by the mediators; and (4) ^3^O_2_ losses resulting from reactive quenching of ^1^O_2_ with solvent or mediator. Simulation results are shown as dashed lines and markers in Fig. 2b,c and resemble the measured data. Deviations result from simplifications of the model, such as not accounting for specific reactivities of the chemically diverse mediators (Methods). Overall, a proper fit of the kinetics to the double parabola in equation (2) (Fig. 2a), the deficiencies in ^3^O_2_ yields and the growing NIR signal beyond the predicted onset potential (Fig. 2b,c) all support the proposed hypothesis that individual Marcus parabolas govern ^3^O_2_ and ^1^O_2_ formation from superoxide.

**Extended Data Fig. 4:**
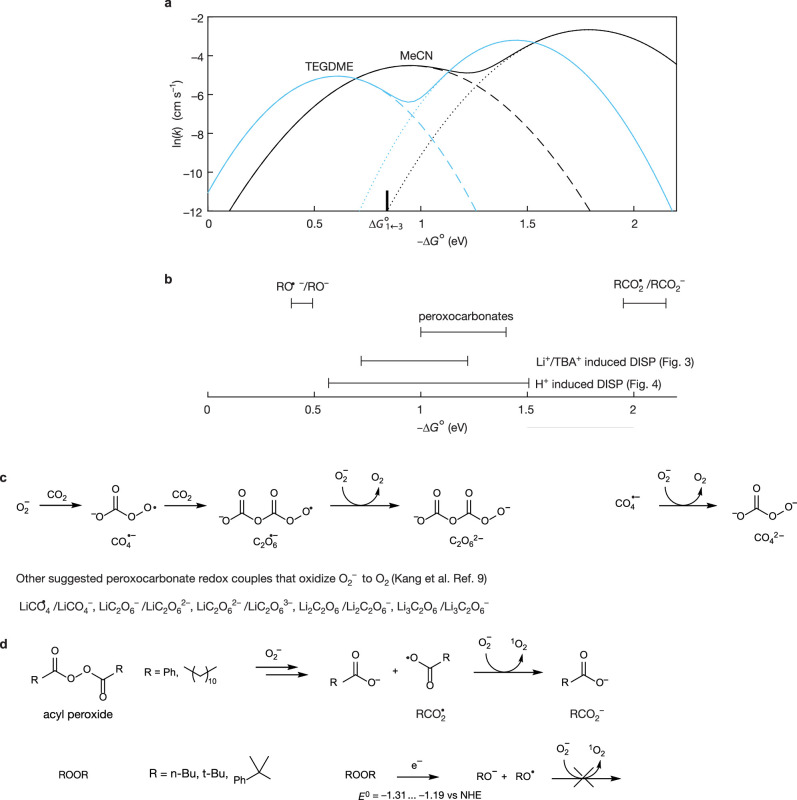
Ranges of driving forces for various systems, where superoxide oxidation occurs. **a**, Comparison of individual kinetic parabolas as determined for mediated superoxide oxidation. Curves for MeCN are the Marcus fits in Fig. 2. For TEGDME the ^3^O_2_ parabola is the Marcus fit to data in Extended Data Fig. 1. The parabola for ^1^O_2_ evolution could not be measured in TEGDME, and is hence constructed using ${\triangle G}_{1\leftarrow 3}^{\circ }$ = 0.84 eV and *Z*_el,1_ = 6.3 × *Z*_el,3_ as determined with MeCN (Fig. 2). **b**, Ranges of driving forces for cation-induced O_2_^–^ disproportionation (DISP) and for the indicated redox couples as defined in c and d. **c**, Commonly suggested peroxocarbonate species that form from O_2_^–^ in contact with CO_2_ (refs. ^41–44^). For example, the superoxo species ${{\rm{CO}}}_{4}^{{\rm{\bullet }}-}$ or ${{\rm{C}}}_{2}{{\rm{O}}}_{6}^{{\rm{\bullet }}-}$ were suggested to be reduced by ${{\rm{O}}}_{2}^{-}$, liberating O_2_. Kang’s group^4^ suggested using DFT further peroxocarbonate redox couples and their redox potentials relative to ${{\rm{O}}}_{2}^{-}$/^3^O_2_ (shown in b). **d**, Reactions of acyl and alkyl peroxides in contact with ${{\rm{O}}}_{2}^{-}$ which were found to form or not to form ^1^O_2_ (refs. ^45,46^) as explicable by their reduction potentials (shown in b).

The adequate fit supports several important conclusions: (1) The prefactors *Z*_el,1_ ≈ 6.3 × *Z*_el,3_ result in substantially higher maximum kinetics for ^1^O_2_ evolution. (2) Driving forces for maximum kinetics are solvent-dependent, as shown by the comparison between values in ether solvent (Fig. 1a; *λ*_3_ = 0.61 eV) and acetonitrile (Fig. 2a; *λ*_3_ = 0.95 eV). This aligns with the prediction^27^ of Marcus and work by Miller^29^. The crossing point of the two parabolas is not at a constant driving force, which benefits ^1^O_2_ for solvents with lower *λ* (Extended Data Fig. 4a). (3) ${E}_{{\genfrac{}{}{0ex}{}{1}{}{\rm{O}}}_{2}/{\rm{superoxide}}}^{\circ }$ should not be considered the threshold above which ^1^O_2_ rather than ^3^O_2_ forms^18,24,26^ as suggested from the electrochemiluminescence literature (Methods) but as a possible onset of ^1^O_2_. Reaching ${E}_{{\genfrac{}{}{0ex}{}{1}{}{\rm{O}}}_{2}/{\rm{superoxide}}}^{\circ }$ per se tells little about the relative ^3^O_2_ and ^1^O_2_ formation kinetics because of the solvent-dependent reorganization energy (Extended Data Fig. 4). Instead, driving forces –Δ*G*° > *λ*_3_ are required to slow ^3^O_2_ formation to benefit ^1^O_2_. (4) In the electrochemiluminescence literature, the entropy change is unknown and typically neglected^28^. Having data that show the peaks for ^3^O_2_ and ^1^O_2_ permits us to determine ${\Delta G}_{1\leftarrow 3}^{\circ }=0.84\,{\rm{eV}}$ and with ${\Delta H}_{1\leftarrow 3}^{\circ }=\,{\Delta G}_{1\leftarrow 3}^{\circ }+T{\Delta S}_{1\leftarrow 3}^{\circ }$ we obtain $T{\Delta S}_{1\leftarrow 3}^{\circ }=-\,0.13\,{\rm{eV}}$. Consequently, ${E}_{{\genfrac{}{}{0ex}{}{1}{}{\rm{O}}}_{2}/{\rm{superoxide}}}^{\circ }={E}_{{\genfrac{}{}{0ex}{}{3}{}{\rm{O}}}_{2}/{\rm{superoxide}}}^{\circ }+0.84\,{\rm{V}}$ can now be given more precisely, rather than with the typically used difference of 0.97 V.

Figure 2a establishes a working curve for an extensive range of Δ*G*°, facilitating an understanding of the behaviour of various important systems. Lewis-acid- and Brønsted-acid-driven superoxide disproportionation are two widely relevant cases, examined in the following sections. More examples of superoxide oxidation, in which an explanation of whether, or not, ^1^O_2_ forms has been unknown, but which can now be explained, are examined in Extended Data Fig. 4 and the Methods. These examples include superoxide in contact with CO_2_ and organic peroxides, with relevance for energy storage and biological systems^1,4,30^.

## Disproportionation in non-aqueous systems

^1^O_2_ is known to cause degradation in non-aqueous alkali metal-O_2_ batteries^3,13,16–19^. The Li–O_2_ battery, for example, operates by reversibly forming lithium peroxide at the positive electrode, O_2_ + 2e^–^ + 2Li^+^ ⇄ Li_2_O_2_. Initially formed LiO_2_ disproportionates, which always results in some ^1^O_2_ according to 2LiO_2_ → Li_2_O_2_ + *x*^3^O_2_ + (1 − *x*)^1^O_2_ (refs. ^13,16–19^). Weak Lewis acids such as tetrabutylammonium (TBA^+^), or other similar cations from ionic liquid electrolytes, are often present in these cells. Despite not driving disproportionation themselves, weak Lewis acids were found to raise the ^1^O_2_ fraction from about 2% in pure Li^+^ electrolyte to around 20% for 1/1 Li^+^/TBA^+^ electrolyte^16^. However, to date, it has been unclear why pure Li^+^ generates ^1^O_2_ at all and why weak Lewis acids should increase the ^1^O_2_ fraction.

**Fig. 3:**
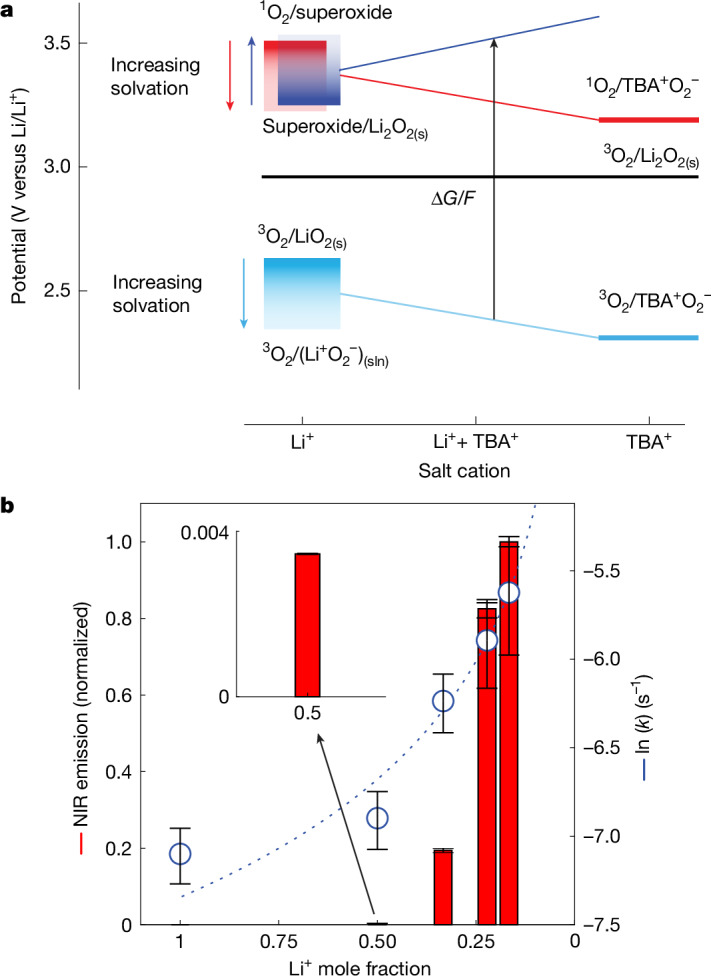
Driving forces, kinetics and ^1^O_2_ formation during Lewis-acid-induced superoxide disproportionation. **a**, Thermodynamics of relevant redox couples for Li^+^-induced superoxide disproportionation as a function of the fractions of Li^+^ and TBA^+^ salt. The gradient boxes and short arrows indicate increasing superoxide solvation with potentials between values relevant for solid LiO_2(s)_ (dark colour), solvated ${({{\rm{Li}}}^{+}{{{\rm{O}}}_{2}}^{-})}_{n\ge 1,({\rm{sln}})}$ clusters, and solvated ${({{\rm{Li}}}^{+}{{{\rm{O}}}_{2}}^{-})}_{({\rm{sln}})}$ (faded colour). LiO_2_ is in all relevant electrolytes at least somewhat soluble as ${({{\rm{Li}}}^{+}{{{\rm{O}}}_{2}}^{-})}_{n\ge 1,({\rm{sln}})}$ clusters. The nature of superoxide shifts from ${({{\rm{Li}}}^{+}{{{\rm{O}}}_{2}}^{-})}_{n\ge 1,({\rm{sln}})}$ towards ${({{\rm{TBA}}}^{+}{{{\rm{O}}}_{2}}^{-})}_{({\rm{sln}})}$ as the cation changes from pure Li^+^ towards pure TBA^+^. The inclined lines indicate the associated shift of the potentials. ${E}_{{\genfrac{}{}{0ex}{}{1}{}{\rm{O}}}_{2}/{\rm{superoxide}}}={E}_{{\genfrac{}{}{0ex}{}{3}{}{\rm{O}}}_{2}/{\rm{superoxide}}}+0.84\,{\rm{V}}$. The driving force $\Delta G=-\,({E}_{{\rm{superoxide}}/{\text{Li}}_{2}{\text{O}}_{2}}-{E}_{{\genfrac{}{}{0ex}{}{3}{}{\rm{O}}}_{2}/{\rm{superoxide}}})F$ grows with the shift of the O_2_/superoxide and superoxide/Li_2_O_2(s)_ potentials. Note that the O_2_ reduction potential changes nonlinearly with the Li^+^:TBA^+^ ratio^32^. **b**, Normalized 1,270 nm NIR emission as a function of Li^+^ mole fraction, which determines the driving force. The markers show superoxide disproportionation kinetics as measured by pressure evolution. The dotted line is a guide to the eye. Glyme served as the electrolyte solvent. Data are presented as mean ± s.d. (*n* ≥ 3). Source Data

**Extended Data Fig. 5:**
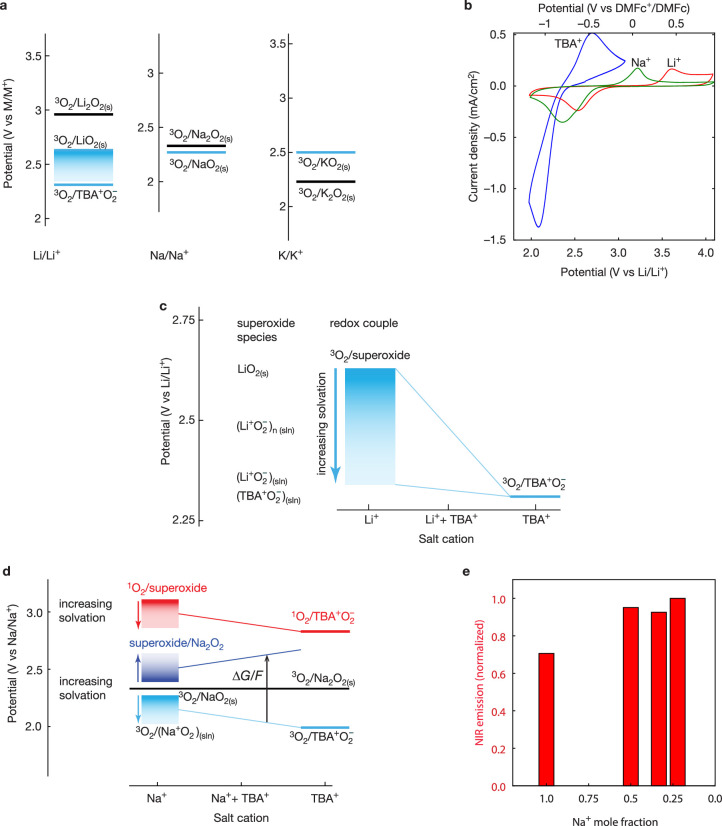
Redox potentials in mixed Li^+^/TBA^+^ and Na^+^/TBA^+^ electrolytes. **a**, Standard potentials of the O_2_/MO_2(s)_ and O_2_/M_2_O_2(s)_ redox couples with M = Li, Na, K as calculated from tabulated formation energies. The common scale is based on their ${E}_{{\rm{M}}/{{\rm{M}}}^{+}}^{\circ }$. **b**, Cyclic voltammograms (scan rate 100 mVs^–1^) in O_2_-saturated tetraglyme containing 100 mM of either TBAClO_4_, LiClO_4_, or NaClO_4_. **c**, Detailed thermodynamics of the ^3^O_2_/superoxide couple as a function of the fractions of Li^+^ and TBA^+^ salt. **d**, Thermodynamics of relevant redox couples for Na^+^-induced ${{\rm{O}}}_{2}^{-}$ disproportionation as a function of the fractions of Na^+^ and TBA^+^ salt. **e**, Normalised 1270 nm NIR emission signal as a function of Na^+^ mole fraction, which determines the driving force. Each bar represents a single measurement. Tetraglyme served as the electrolyte solvent.

Figure 3a shows the thermodynamics of the relevant redox couples as a function of the Li^+^ and TBA^+^ salt fractions in glyme electrolyte. Figure 3b shows the NIR emission intensities on adding KO_2_ as a superoxide source into these electrolytes. The thermodynamics are explored in further detail in the Methods and Extended Data Fig. 5 and are summarized here. Li_2_O_2_ is insoluble, and the potential ${E}_{{\genfrac{}{}{0ex}{}{3}{}{\rm{O}}}_{2}/{{\rm{Li}}}_{2}{{\rm{O}}}_{2}({\rm{s}})}$, therefore, fixed (Fig. 3a, black line). However, superoxide is appreciably soluble in non-aqueous electrolytes^31–33^: species include ${({{\rm{Li}}}^{+}{{{\rm{O}}}_{2}}^{-})}_{n\ge 1,({\rm{sln}})}$ clusters or ion pairs, where (sln) denotes solvate species. Solid LiO_2(s)_ has the lowest Gibbs free energy, and increasingly solvated species are increasingly less stable^32–34^. The ^3^O_2_/superoxide potentials are, therefore, within the upper limit of ${E}_{{\genfrac{}{}{0ex}{}{3}{}{\rm{O}}}_{2}/{\rm{Li}}{{\rm{O}}}_{2}({\rm{s}})}$ and the lower limit of ${E}_{{\genfrac{}{}{0ex}{}{3}{}{\rm{O}}}_{2}/{({{\rm{Li}}}^{+}{{\rm{O}}}_{2}^{-})}_{({\rm{sln}})}}$ (Fig. 3a, light-blue gradient-coloured box). When TBA^+^ is present, the lower limit extends to ${E}_{{\genfrac{}{}{0ex}{}{3}{}{\rm{O}}}_{2}/{({{\rm{TBA}}}^{+}{{\rm{O}}}_{2}^{-})}_{({\rm{sln}})}}$.

The superoxide/Li_2_O_2(s)_ couple acts as the oxidant during superoxide disproportionation. ${E}_{{\rm{superoxide}}/{{\rm{Li}}}_{2}{{\rm{O}}}_{2(\text{s})}}$ exceeds ${E}_{{{}^{1}{\rm{O}}}_{2}/{\rm{superoxide}}}$ already in pure Li^+^ electrolyte, in which the superoxide species are solvated, and therefore explains why an onset of ^1^O_2_ is observed. ^1^O_2_ is detected in pure Li^+^ electrolyte using more sensitive chemical trapping^16^. Addition of TBA^+^ favours more solvated species, which also explains why TBA^+^ increases the fraction of ^1^O_2_. In line with this, Fig. 3b shows steeply increasing ^1^O_2_ evolution as the TBA^+^ mole fraction increases, along with faster kinetics.

## Proton-induced disproportionation

**Fig. 4:**
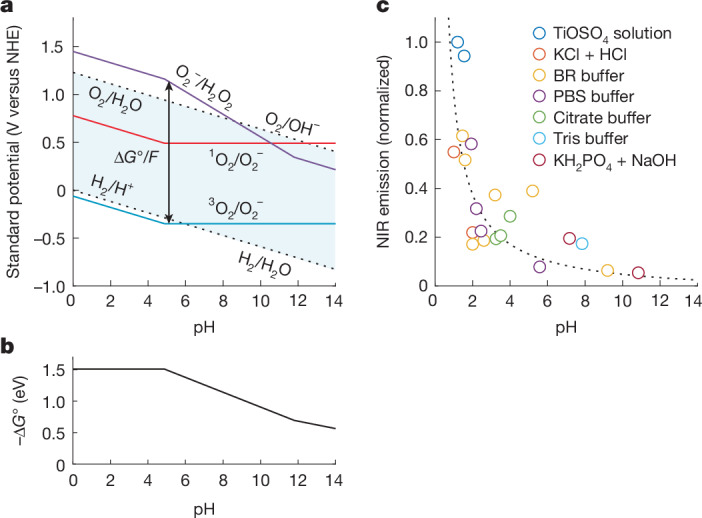
Driving forces and NIR emission during proton-induced superoxide disproportionation. **a**, Pourbaix diagram showing the pH-dependent stability range of aqueous electrolyte and the standard potentials ${E}^{\circ }$ of the indicated redox couples on the normal hydrogen electrode (NHE) scale^15^. The kinks in the curves arise from the p*K*_a_ values of H_2_O_2_ (p*K*_a_ = 11.7) and HO_2_ (p*K*_a_ = 4.8). For simplicity, O_2_^–^/H_2_O_2_ is written while the pH-dependent (de)protonated species are meant. ${E}_{{\genfrac{}{}{0ex}{}{1}{}{\rm{O}}}_{2}/{{{\rm{O}}}_{2}}^{-}}^{\circ }={E}_{{\genfrac{}{}{0ex}{}{3}{}{\rm{O}}}_{2}/{{\rm{O}}}_{2}^{-}}^{\circ }+0.84\,{\rm{V}}.$ **b**, The pH-dependent driving force for superoxide disproportionation is $-\Delta {G}^{\circ }=({E}_{{{\rm{O}}}_{2}^{-}/{\text{H}}_{2}{\text{O}}_{2}}^{\circ }-{E}_{{\genfrac{}{}{0ex}{}{3}{}{\rm{O}}}_{2}/{{\rm{O}}}_{2}^{-}}^{\circ })F$ as indicated by the vertical arrows in **a**. **c**, ^1^O_2_-specific NIR emission at 1,270 nm as a function of pH. Each marker represents a single measurement. The dotted line is a guide to the eye. PBS, phosphate-buffered saline. Source Data

We next investigated how pH-related changes in driving force affect the ^1^O_2_ formation from proton-induced superoxide disproportionation. This is particularly relevant for living organisms, in which superoxide is found in organelles with pH levels ranging between 4.7 and 8 (ref. ^35^), as well as in aqueous electrocatalysis^10^. Figure 4a shows the Pourbaix diagram with the potentials of relevant redox couples as a function of pH. Again, the driving force for disproportionation is the difference between the ^3^O_2_/superoxide and superoxide/peroxide couple, marked with the vertical arrows. It grows from about 0.5 eV at pH 14 to about 1.5 eV below pH 4.8 (Fig. 4b). The oxidant potential exceeds ${E}_{{\genfrac{}{}{0ex}{}{1}{}{\rm{O}}}_{2}/{{\rm{O}}}_{2}^{-}}^{\circ }$ at pH about 10.5, in which an onset of ^1^O_2_ formation can be expected.

Figure 4c shows the ^1^O_2_-specific NIR signal at 1,270 nm on proton-induced superoxide disproportionation over a pH range from about 1 to 10.8. We exposed KO_2_ to various buffer solutions under vigorous stirring and recorded the NIR signal (Methods). A strongly increasing NIR signal with decreasing pH is in accord with the increasing driving force. A pH of around 11 giving low but non-negligible ^1^O_2_ yields is consistent with reliable theoretical^17^ and experimental works^14^ on the reaction ${{\rm{HO}}}_{2}+{{\rm{O}}}_{2}^{-}\to {{\rm{O}}}_{2}+{{\rm{O}}}_{2}^{-}$, which found ^1^O_2_ evolution only about 0.3 eV endoergic and around 0.2% ^1^O_2_ production on exposing KO_2_ to H_2_O_2_ as the proton source.

## Conclusions

We found that the driving force for superoxide oxidation to ^3^O_2_ and ^1^O_2_ is the common descriptor that determines the spin state, following individual Marcus normal and inverted region behaviour. ^1^O_2_ can become significant only because the kinetics for ^3^O_2_ evolution slows down in its inverted region. The results help clarify previously inconclusive findings about ^1^O_2_ formation from superoxide, including through interaction with chemical oxidants, and proton and Lewis-acid-driven disproportionation. For disproportionation, the results explain the increasing ^1^O_2_ formation with stronger Brønsted and weaker Lewis acidity, respectively, because of their impact on driving forces. Increasing ^1^O_2_ yield with lower pH aligns with higher driving forces as the pH decreases. This corresponds with higher and lower pH in respiratory (mitochondria) and degenerative organelles (lysosomes)^35^, respectively, in which ^1^O_2_ must be avoided or may even be beneficial. This connection between pH and ^1^O_2_ formation may well have been a so far unrecognized evolutionary driver for the pH found in organelles. In human-made redox systems, in which ^1^O_2_ is, in most cases, detrimental and damaging, strategies to suppress ^1^O_2_ should aim at reducing the driving forces for superoxide oxidation, increasing the reorganization energy, or avoiding situations in which superoxide disproportionates. We discuss the wider relevance for oxygen redox systems in life sciences and energy, and for the electrogeneration of excited species more generally in the Methods. The findings offer insights into understanding and controlling spin states and kinetics in oxygen redox chemistry, with implications for fields such as the life sciences and energy storage.

## Methods

### Materials

All chemicals were from Sigma Aldrich unless indicated differently. Potassium superoxide (KO_2_), potassium perchlorate (KClO_4_, ≥99.99%), lithium bis(trifluoromethanesulfonyl)imide (LiTFSI, 99.99%) and tetrabutylammonium bis(trifluoromethanesulfonyl)imide (TBATFSI, ≥99.0%) were dried under reduced pressure for 24 h at 100 °C. Decamethylferrocene (DMFc), tris[4-(diethylamino)phenyl]amine (TDPA), *N,N,N*′*,N*′-tetramethyl-*p*-phenylenediamine (TMPD), ferrocene (Fc), ferrocenium tetrafluoroborate (FcBF_4_), 9,10-diphenylanthracene (DPA), 1,4-bis(diphenylamino)benzene (DPAB), thianthrene (ThA), 1,4-dimethoxyanthracene (DMeOA, Acros Organics), 1,4-di-tert-butyl-2,5-dimethoxybenzene (tMeOB, BLDpharm) and 5,10-dihydro-5,10-dimethylphenazine (DMPZ, TCI chemicals) were used as received. *N*-methyl phenothiazine (MPT), 1,4-diazabicyclo[2.2.2]octane (DABCO) were sublimated. 18-Crown-6 and tetrafluorobenzoquinone (F_4_BQ) were recrystallized from ethanol. Tetraethylene glycol dimethyl ether (TEGDME, ≥99%) was dried over lithium, distilled under vacuum and further stored over activated 3 Å molecular sieves. Acetonitrile (MeCN, 99.8% anhydrous) was stored over activated molecular sieves. Both solvents had a water content below 5 ppm as determined by Karl–Fischer titration (Mettler Toledo). All non-aqueous experiments were performed in an Ar-filled glovebox (Vigor) or hermetically sealed setups without air exposure. The structures of the mediators and their redox potentials are shown in Extended Data Fig. 2.

### Electrochemistry and mediator oxidation

Electrochemistry experiments were performed using a BioLogic potentiostat (SP-300 and MPG-2). Cyclic voltammetry was performed with a glassy carbon disk as the working electrode and a glassy carbon rod as the counter electrode in a one-compartment glass cell. Partially delithiated Li_(1–*x*)_FePO_4_ (LFP, MTI), separated by a Vycor glass frit, was used as the reference electrode. DMFc/DMFc^+^ was used as the internal standard and converted using $${E}_{{\rm{DMFc}}/{{\rm{DMFc}}}^{+}}^{\circ }={E}_{{\rm{Li}}/{{\rm{Li}}}^{+}}^{\circ }+3.16\,{\rm{V}}={E}_{{\rm{K}}/{{\rm{K}}}^{+}}^{\circ }$$$$+3.02\,{\rm{V}}.$$ DMeOA, tMeOB, DPA and ThA were electrochemically oxidized in an H-cell. The oxidation compartment contained a Pt working electrode, the reference electrode and 5 ml MeCN containing 2 mM RM and 10 mM KClO_4_. The reduction compartment contained Ni foam as a counterelectrode with 5 ml of 100 mM 1,4-benzoquinone (sublimed) in MeCN. A K^+^ selective ion-exchange membrane separated the two compartments. RMs were oxidized galvanostatically to 80% of their total capacity. DMFc, TDPA, TMPD, DMPZ, MPT and DPAB were oxidized using 1 equiv. NOBF_4_ in MeCN. After 3 h stirring, they were precipitated with cold diethyl ether, filtered and dried under a vacuum at 30 °C for 12 h. F_4_BQ is the oxidized form itself.

### Measurements of kinetics

Kinetics of mediated superoxide oxidation were measured using UV–Vis spectroscopy using an Avantes AvaSpec-HSC spectrometer with AVALIGHT-DH-S-BAL light source and fibre optics to perform measurements inside the glove box. Pure KO_2_ powder was pressed into about 0.5 mm thick pellets using a 7 mm die set and a hand press (PIKE). In a 10-mm quartz cuvette (Hellma), a KO_2_ pellet was placed in a polytetrafluoroethylene frame for alignment, followed by a magnetic stirring bar and then the cuvette was sealed with a gas-tight injection lid. RM^ox^ solution containing 10 mM KClO_4_ was then injected using a gas-tight syringe (Hamilton). Consumption of RM^ox^ was followed except for F_4_BQ, in which formation of RM^red^ was followed (Extended Data Fig. 7). Data and error bars are presented as mean ± s.d. (*n* ≥ 3). Error bars appear asymmetric on an ln(*k*) scale. Repetitions mean that each time a new portion of RM^ox^ was produced by electrochemically oxidizing a portion of RM^red^ or by dissolving the chemically produced oxidized form. Differences in the magnitude of the error bars among mediators arise from their large chemical diversity and specific reactivities. For example, Fc^+^ and reduced quinones react with O_2_, or high-voltage RM^ox^ shows limited long-term stability in the electrolyte. For the latter, it was checked that degradation was at least several times slower than the oxidation of KO_2_.

Kinetics of Li^+^-induced superoxide disproportionation in TEGDME were measured by placing KO_2_ powder in a closed reaction vessel equipped with a pressure sensor (Omega, PAA35X) and injecting the Li^+^ electrolyte using a syringe through a septum (Extended Data Fig. 8).

### ^3^O_2_ yields and 1,270 nm emission measurements

**Extended Data Fig. 6:**
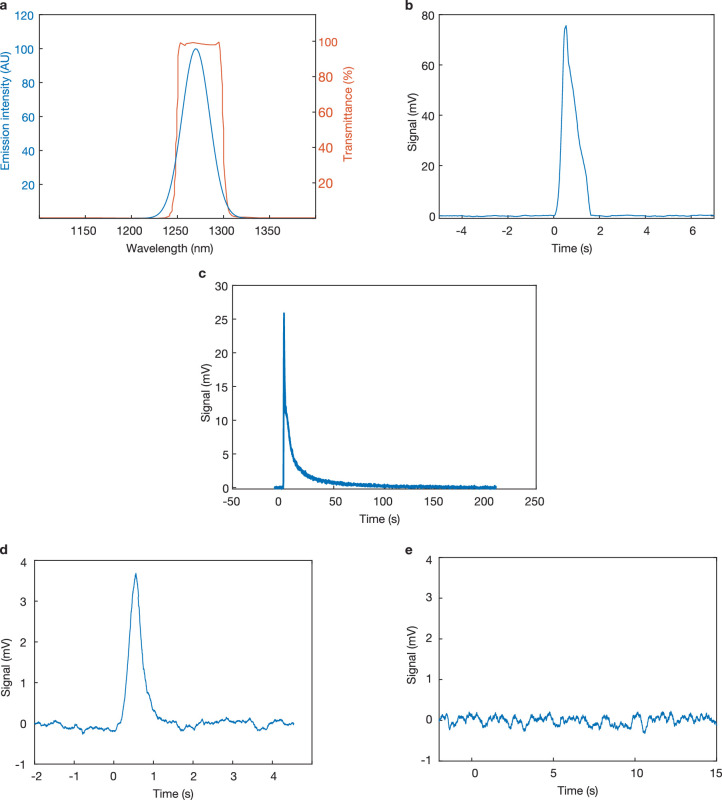
NIR emission to measure ^1^O_2_ yields. **a**, ^1^O_2_ NIR emission spectrum as reported in the literature^12^ and transmission of the used bandpass filter. **b**, Representative output signal upon injecting RM^ox^ solution onto KO_2_ powder. The integral of the recorded signal was used to determine the ^1^O_2_ yields. The example shown is with MPT. **c**, Representative output signal of the NIR detector upon injecting Li^+^ electrolyte solution onto KO_2_ powder. The setup was similar to the one shown in Extended Data Fig. 4, except that a cuvette replaced the glass vial and the NIR detector was placed next to it. The example shown is with a Li^+^ mole fraction of 0.166. **d**, Representative NIR signal upon injecting H^+^ solution containing buffer onto KO_2_ powder. The example shown is with BR buffer at pH 2.2. **e**, Output signal of the NIR detector upon injecting H_2_SO_4_ (pH 1.58) solution without any buffer onto KO_2_ powder.

**Extended Data Fig. 7:**
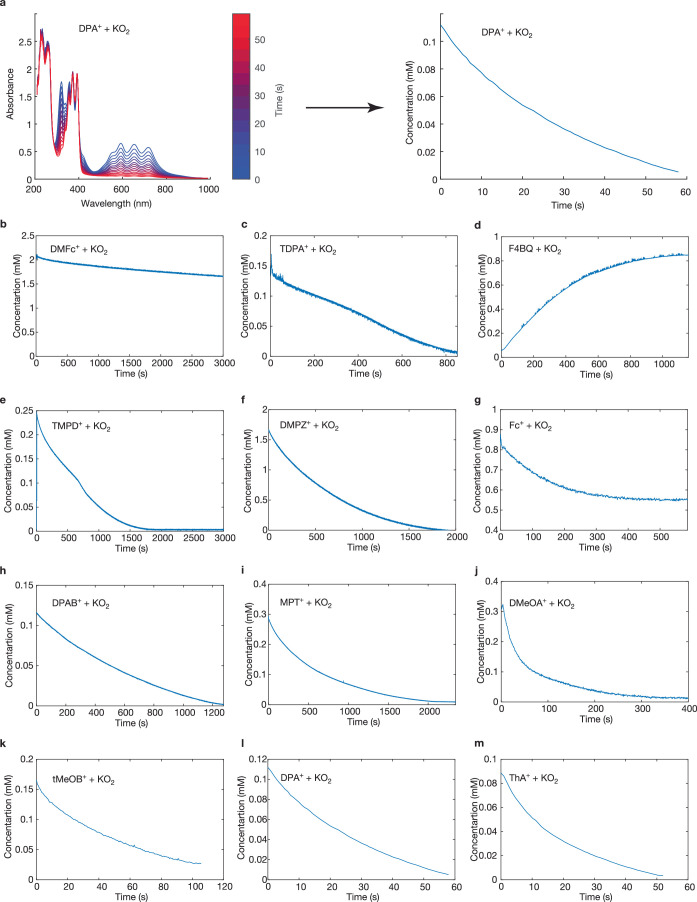
Measuring KO_2_ oxidation kinetics using UV-Vis absorbance. **a**, Exemplary evolution UV-Vis spectra of DPA and ThA. Concentration versus time for the indicated RM^ox^ in contact with KO_2_. **b**,**c**,**e–m**, RM^ox^ concentration versus time. For F4BQ (**d**), concentration versus time for RM^red^.

**Extended Data Fig. 8:**
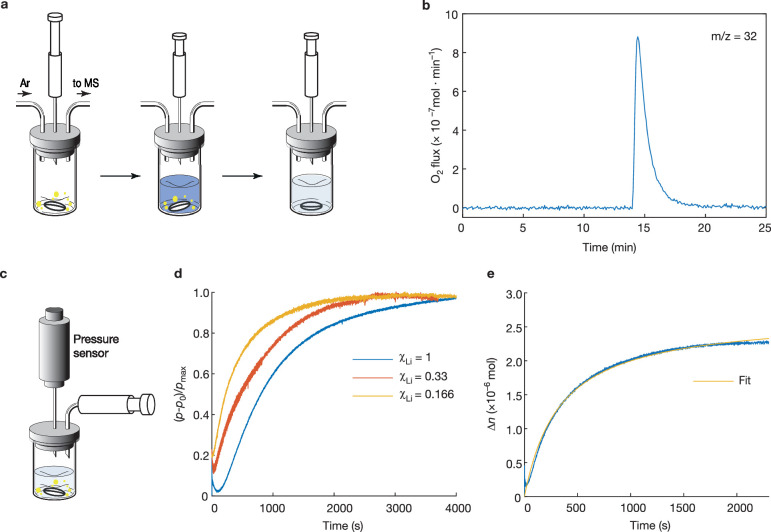
Measuring ^3^O_2_ yields and disproportionation kinetics. **a**, Schematic set-up of the sample vial used for mass spectrometry and sequence of the solution addition during the ^3^O_2_ yield measurement. **b**, O_2_ evolution versus time upon immersing KO_2_ powder in RM^ox^ solution. The example shown is with DMPZ. **c**, Schematic of the set-up to measure disproportionation kinetics by pressure changes. **d**, Pressure evolution from three different cases where Li^+^/(Li^+^+TBA^+^) mole fractions are 1, 0.33 and 0.166. **e**, Evolved moles versus time for superoxide disproportionation as measured by the pressure *p*. Fitting is done with *p*(*t*) = *p*_0_ + Δ*p*(1–exp(–*kt*)). The example is shown for the case with the mole fraction Li^+^/(Li^+^+TBA^+^) = 0.166. Panel **a** adapted with permission from ref. ^64^, American Chemical Society.

^3^O_2_ yields on mediated superoxide oxidation were measured using mass spectrometry, as detailed previously^30^. The RM^ox^ solutions were injected using a gas-tight syringe, and the measurement continued until the O_2_ signal ceased. We used the ^1^O_2_-specific NIR emission at 1,270 nm from the decay of ^1^O_2_ to ^3^O_2_ to determine ^1^O_2_ yields and lifetimes as detailed previously^30^. The signal was recorded from the detector using an oscilloscope (Pico Technology) and at a gain of 820 V (control voltage). Extended Data Fig. 6 shows examples of the recorded signal during mediated oxidation, as well as Li^+^- and H^+^-induced disproportionation.

### From oxidation rates to ^3^O_2_ yields and NIR emission intensities

We use the yields of ^3^O_2_/RM^ox^ and the normalized NIR intensities in Fig. 2b,c to prove that the two kinetic parabolas correspond to ^3^O_2_ and ^1^O_2_ evolution. To rationalize their assignment, we use a minimal model to calculate expected ^3^O_2_/RM^ox^ and NIR emission intensities based on formation rates and ^1^O_2_ decay processes. We considered the following processes: (1) ^3^O_2_ and ^1^O_2_ formation rates (*k*_3_ and *k*_1_) as given by the two kinetic parabolas in Fig. 2a; (2) physical and reactive ^1^O_2_ quenching by the solvent; (3) physical and reactive ^1^O_2_ quenching by the reduced mediators RM^red^. Note that the same processes with RM^ox^ are typically negligible in comparison because of the electron demand of these processes^36^; and (4) ^3^O_2_ losses (^3^O_2_/RM^ox^ < 1) resulting from reactive quenching of ^1^O_2_ with solvent or mediator.

Losses in ^3^O_2_/RM^ox^ in Fig. 2b result from incomplete physical quenching of ^1^O_2_ to ^3^O_2_ due to reactions of ^1^O_2_ with electrolyte or RM. Hence, we start by considering the decay routes. There are multiple decay routes, as shown in Extended Data Fig. 3f, and only a small fraction of the total ^1^O_2_ can be detected by the NIR emission at 1,270 nm (refs. ^12,37^). First, interactions of ^1^O_2_ with solvent will result in electronic-to-vibrational (e–v) deactivation and reactions with the solvent. The first-order ^1^O_2_ decay rate constant *k*_D_ = *k*_d,r_ + *k*_d_ is composed of a reactive fraction *k*_d,r_ and a non-reactive fraction *k*_d_. Second, electron-rich species, such as reduced mediators, exert charge transfer quenching; however, they may also react with the electrophilic ^1^O_2_ along pathways depending on the particular chemistry^36^. The rate constant *k*_Q_ = *k*_q,r_ + *k*_q_ is equally composed of a reactive fraction *k*_q,r_ and a non-reactive fraction *k*_q_. Third, radiative decay of ^1^O_2_ to ^3^O_2_ with the emission of a 1,270 nm photon.

We measured the rate constants *k*_D_ and *k*_Q_ using the luminescence lifetime. An O_2_-saturated solution containing Rose Bengal (absorbance of about 0.1 in a 1 cm fluorescence cuvette) was illuminated using a pulsed Coherent OBIS 561 nm laser. The lifetime (*τ*) and the decay constant (*k* = 1/*τ*) of ^1^O_2_ were measured using the NIR detector. *τ* was obtained by fitting the decay profile with exp(–*t*/*τ*) (Extended Data Fig. 3a). *τ* in the absence of a quencher relates to the solvent quenching rate constant *k*_D_. *k*_Q_ is obtained by plotting 1/*τ* against the RM^ox^ concentration ${c}_{{{\rm{RM}}}^{{\rm{ox}}}}$ (Extended Data Fig. 3b,c). Similar to previously reported quenchers^12^, *k*_Q_ decreases with ${E}_{{\text{RM}}^{\text{ox}/\text{red}}}^{\circ }$ following a trendline with a slope of about –10^3.5^ V^−1^. The logarithmic dependence of the non-radiative fraction *k*_q_ is explicable by the required partial charge transfer from the e^–^-rich quencher to the ^1^O_2_, which makes quenchers with lower redox potentials more efficient^12^. We consider that the reactive fraction *k*_q,r_ depends similarly on redox potential, given that the addition reactions equally require charge transfer from the substrate to ^1^O_2_ (refs. ^12,36^). The scattering of *k*_Q_ around the trendline arises from the large chemical diversity of the used mediators. We used both the trendline and the individual values of *k*_Q_ for the prediction of NIR intensities in Fig. 2c. As for the fraction of reactive deactivation, *k*_d,r_/*k*_D_ and *k*_q,r_/*k*_Q_, these can be determined from ^3^O_2_ consumption^12^, which we do below when simulating values of ^3^O_2_/RM^ox^.

The surface area *A* of the KO_2_ powder, needed for the calculations, was determined by analysing optical images (Extended Data Fig. 3d). KO_2_ powder was dispersed in TEGDME, sonicated, a drop was placed between a microscope slide and a cover slip and sealed air-tight. Images were acquired with transmitted light on a Nikon Ti2E-01 inverted microscope using a Plan Apo λ 40×/0.95 DIC (Differential Interference Contrast) air PFS (Perfect Focus System) objective lens, resulting in a pixel size of 0.183 μm. The images were analysed using the ilastik pixel classification and object classification workflows (https://www.ilastik.org/), resulting in a histogram of particle sizes (Extended Data Fig. 3e). The surface area was calculated by assuming spherical particles, yielding *A* = 0.23 ± 0.04 m^2^ g^–1^.

As quantification of absolute ^1^O_2_ yields from NIR emission is not straightforward, we normalized the values. The NIR intensity at time *t* after bringing KO_2_ in contact with RM^ox^ will be proportional to the ^1^O_2_ concentration, $${I}_{1,270{\rm{nm}}}(t)\propto {c}_{{\genfrac{}{}{0ex}{}{1}{}{\rm{O}}}_{2}}(t)$$. The ^1^O_2_ formation rate by mediated KO_2_ oxidation is $${\nu }_{{\genfrac{}{}{0ex}{}{1}{}{\rm{O}}}_{2}}(t)=A\cdot {k}_{1}\cdot {c}_{{{\rm{RM}}}^{{\rm{ox}}}}(t)$$, with *k*_1_ being the rate constant as given in Fig. 2a. *A* is constant because the experiments were performed with a large excess of KO_2_ over RM^ox^. $${c}_{{{\rm{RM}}}^{{\rm{ox}}}}$$ and $${c}_{{{\rm{RM}}}^{{\rm{red}}}}$$ are given by $${c}_{{{\rm{RM}}}^{{\rm{ox}}}}(t)={c}_{{{\rm{RM}}}^{{\rm{ox}}}}(0)\cdot {{\rm{e}}}^{-{k}_{{\rm{tot}}}\cdot A\cdot t}$$ and $${c}_{{{\rm{RM}}}^{{\rm{red}}}}(t)={c}_{{{\rm{RM}}}^{{\rm{ox}}}}(0)-{c}_{{{\rm{RM}}}^{{\rm{ox}}}}(t)+$$$${c}_{{{\rm{RM}}}^{{\rm{red}}}}(0)-{\nu }_{{\genfrac{}{}{0ex}{}{1}{}{\rm{O}}}_{2},{\rm{q}},{\rm{r}}}(t)\cdot t$$, where *k*_tot_ = *k*_1_ + *k*_3_. The term $${\nu }_{{\genfrac{}{}{0ex}{}{1}{}{\rm{O}}}_{2},{\rm{q}},{\rm{r}}}(t)$$ is the rate at which ^1^O_2_ reacts with RM^red^ as detailed below. ^1^O_2_ formation and decay will balance^37^, which results in the presence of solvent and mediator quenching in $${c}_{{\genfrac{}{}{0ex}{}{1}{}{\rm{O}}}_{2}}(t)={\nu }_{{\genfrac{}{}{0ex}{}{1}{}{\rm{O}}}_{2}}(t)/({k}_{{\rm{d}}}+{k}_{{\rm{Q}}}\cdot {c}_{{{\rm{RM}}}^{{\rm{red}}}}(t))$$. *k*_Q_ is either the measured value for the particular mediator or the trendline in Extended Data Fig. 3c. Finally, analogous to the experiment where we integrate the NIR signal until it ceases, we arrive at the expected NIR emission:

3 $${I}_{1270{\rm{nm}},{\rm{cum}}}\propto {\int }_{0}^{\infty }\,\frac{A\cdot {k}_{1}\cdot {c}_{{{\rm{RM}}}^{{\rm{ox}}}}(t)}{{k}_{{\rm{D}}}+{k}_{{\rm{Q}}}\cdot {c}_{{{\rm{RM}}}^{{\rm{red}}}}(t)}{\rm{d}}t.$$

To simulate losses of ^3^O_2_ (^3^O_2_/RM^ox^ < 1 in Fig. 2b), we used the rate of reactive ^1^O_2_ decay ${\nu }_{{\genfrac{}{}{0ex}{}{1}{}{\rm{O}}}_{2},{\rm{r}}}(t)=({k}_{{\rm{d}},{\rm{r}}}+{k}_{{\rm{q}},{\rm{r}}}\cdot {c}_{{{\rm{RM}}}^{{\rm{red}}}}(t))\cdot {c}_{{\genfrac{}{}{0ex}{}{1}{}{\rm{O}}}_{2}}(t)$ to define *f*_r_ as the reactive fraction of the total ^1^O_2_ decay rate

4 $${f}_{{\rm{r}}}=\frac{{k}_{{\rm{d}},{\rm{r}}}+{k}_{{\rm{q}},{\rm{r}}}\cdot {c}_{{{\rm{RM}}}^{{\rm{red}}}}(t)}{{k}_{{\rm{D}}}+{k}_{{\rm{Q}}}\cdot {c}_{{{\rm{RM}}}^{{\rm{red}}}}(t)}.$$

${\nu }_{{}^{1}{\rm{O}}_{2},{\rm{r}}}(t)$ equals the total mediated ^1^O_2_ formation rate times *f*_r_: ${\nu }_{{\genfrac{}{}{0ex}{}{1}{}{\rm{O}}}_{2},{\rm{r}}}(t)={f}_{{\rm{r}}}\cdot A\cdot {k}_{1}\cdot {c}_{{{\rm{RM}}}^{{\rm{ox}}}}(t)$. As some of the RM^red^ reacts, its loss is accounted for using ${\nu }_{{\genfrac{}{}{0ex}{}{1}{}{\rm{O}}}_{2},{\rm{q}},{\rm{r}}}(t)={k}_{{\rm{q}},{\rm{r}}}\cdot {c}_{{{\rm{RM}}}^{{\rm{red}}}}(t)\cdot {c}_{{\genfrac{}{}{0ex}{}{1}{}{\rm{O}}}_{2}}(t)={f}_{{\rm{q}},{\rm{r}}}\,\cdot A\cdot {k}_{1}\cdot {c}_{{{\rm{RM}}}^{{\rm{ox}}}}(t)$, where ${f}_{{\rm{q}},{\rm{r}}}=\,{k}_{{\rm{q}},{\rm{r}}}\cdot {c}_{{{\rm{RM}}}^{{\rm{red}}}}(t)/({k}_{{\rm{D}}}+{k}_{{\rm{Q}}}\cdot {c}_{{{\rm{RM}}}^{{\rm{red}}}}(t))$. Calculations of *f*_r_, *f*_q,r_ and ${c}_{{{\rm{RM}}}^{{\rm{red}}}}(t)$ were iterated until convergence was achieved. Finally, ^3^O_2_ yields are

5 $${{}^{3}{\rm{O}}}_{2}/{{\rm{RM}}}^{{\rm{ox}}}=1-{\int }_{0}^{\infty }{f}_{{\rm{r}}}\cdot A\cdot {k}_{1}\cdot {c}_{{{\rm{RM}}}^{{\rm{ox}}}}(t){\rm{d}}t/{\int }_{0}^{\infty }A\cdot {k}_{{\rm{tot}}}\cdot {c}_{{{\rm{RM}}}^{{\rm{ox}}}}(t){\rm{d}}t.$$

The fractions of reactive deactivation, *k*_d,r_/*k*_D_ and *k*_q,r_/*k*_Q_ were obtained by fitting the measured values of ^3^O_2_/RM^ox^ in Fig. 2b with the simulated ones from equation (5). The simulated ^3^O_2_/RM^ox^ are plotted as the dashed line in Fig. 2b.

Deviations between simulated and measured values may result from the simplicity of the model. The main simplification is as follows: (1) *k*_Q_ was measured in homogeneous solutions of RM^red^ and the model calculates bulk concentrations ${c}_{{{\rm{RM}}}^{{\rm{ox}}}}$ and ${c}_{{{\rm{RM}}}^{{\rm{red}}}}$, but during the heterogenous KO_2_ oxidation, bulk and surface concentrations of RM^ox^ and RM^red^ differ. (2) We fitted a common fraction of mediator reactivity (*k*_q,r_/*k*_Q_) for all mediators, causing *k*_q,r_ and *k*_Q_ to decrease exponentially with growing ${E}_{{\text{RM}}^{\text{ox}/\text{red}}}^{\circ }$. As a trend, this is justified given that both reaction and charge transferquenching require e^–^ transfer, but individual reactivity will vary because of the large chemical diversity of mediators^36^. (3) We did not account for quenching by O_2_ and ${{\rm{O}}}_{2}^{-}$. (4) Some RMs show further reactivities, which can cause ^3^O_2_ loss: Fc^+^ reacts with O_2_ (ref. ^38^), TDPA gets in contact with O_2_ spontaneously oxidized to TDPA^+^ and TDPA^2+^ (ref. ^18^), and reduced quinones bind to O_2_ (ref. ^39^).

### Proton-induced disproportionation

Six types of buffers were used for proton-induced superoxide disproportionation. Britton–Robinson (BR) buffers were prepared with 0.1 M acetic acid, 0.1 M boric acid and 0.1 M phosphoric acid. NaOH (1 M) and HCl (1 M) were used to adjust the pH. Phosphate-buffered saline (PBS) solution and aqueous PBS powder solution (for pH 7.4) were added and the pH was adjusted using 1 M NaOH or 1 M HCl. Citrate buffers were prepared using citric acid and trisodium citrate dihydrate. To adjust the pH, we varied the concentrations of citric acid and trisodium citrate dihydrate. Acidic buffers of KCl and HCl (pH 1 and 2) were prepared by mixing 0.2 M KCl with 0.2 M HCl and adjusting the volume. Neutral to basic buffers of KH_2_PO_4_/NaOH (pH 7.1 and 10.8) were prepared by mixing 0.1 M KH_2_PO_4_ with 0.1 M NaOH and adjusting the volume accordingly. To trap the hydrogen peroxide (H_2_O_2_) produced, to eliminate the possibility of forming ^1^O_2_ by peroxoacids^40^, titanium(IV)oxysulfate solution (TiOSO_4_, 15 wt% in dilute sulfuric acid), 1 M NaOH was used to adjust the pH of TiOSO_4_ solutions to pH 1.2 and 1.55.

Figure 4c shows ^1^O_2_ yields still increasing when the pH is below 4.8, the p*K*_a_ of O_2_^−^. KO_2_ hydrolysis increases the pH in aqueous media according to KO_2_ + H_2_O → HO_2_ + K^+^ + OH^–^. Buffer capacities were selected so that the amount of KO_2_ did not significantly affect the resulting pH after the reaction. However, despite the buffers, the local pH at the reaction site will be higher than the average. The importance of buffering for a local pH close to the average is evident in control experiments without a buffer. Even at a pH of 1.5, we could not detect ^1^O_2_ in an unbuffered H_2_SO_4_ solution, whereas we could in the buffered one (Extended Data Fig. 6e). Therefore, the ^1^O_2_ yields in Fig. 4c result from a higher local pH.

### Driving forces on superoxide oxidation

Superoxide experiences a broad range of oxidizing conditions to liberate oxygen, but explanations for why and to what extent certain oxidizing redox couples evolve ^1^O_2_ have been unknown. Extended Data Fig. 4 shows the driving forces for superoxide oxidation with various redox couples. The driving forces are shown in comparison with the Marcus kinetic parabola in ether and acetonitrile solvent from Figs. 1 and 2. Li^+^- and H^+^-induced disproportionation are shown in Figs. 3 and 4. The other examples we discuss in Extended Data Fig. 4 arise from superoxide in contact with CO_2_ or organic peroxides, with relevance for energy storage and biology^1,30,41^.

Considering CO_2_ first, we have previously shown that CO_2_ in contact with O_2_^−^ yields ^1^O_2_, but the energetics were unknown^30^. CO_2_ in contact with O_2_^−^ is known to form peroxomonocarbonates and peroxodicarbonates by repeated uptake of CO_2_ by O_2_^−^. Intermediate peroxocarbonate species may be reduced by O_2_^−^, which releases O_2_ (refs. ^41–44^). However, the O_2_ spin state has previously not been considered. Extended Data Fig. 4c shows likely redox couples of oxidized/reduced peroxocarbonate species, but their redox potentials are not established experimentally. A previous study has shown, using density functional theory (DFT), that depending on the cation present and the solvent, the particular peroxocarbonate redox couples that oxidize O_2_^−^ to O_2_ differ^4^, but likely involve CO_4_^•–^/CO_4_^2–^, LiCO_4_^•^/LiCO_4_^–^, Li_2_C_2_O_6_/Li_2_C_2_O_6_^–^ or Li_3_C_2_O_6_/Li_3_C_2_O_6_^–^. The calculations have shown reaction energies relative to the O_2_^−^/^3^O_2_ between about 1 and 1.4 eV. These driving forces, hence, explain ^1^O_2_ formation from CO_2_ in contact with O_2_^−^ (Extended Data Fig. 4b).

^1^O_2_ formation has been examined for exposure of superoxide to organic peroxides, given their occurrence in biological systems^45^. No reaction was observed with alkyl peroxides, whereas acyl peroxides yielded ^1^O_2_ (refs. ^45,46^); however, this has not been connected with driving forces for superoxide oxidation. Nucleophilic attack of O_2_^−^ on acyl peroxides forms an acyl radical and carboxylate (Extended Data Fig. 4d). The potential for the acyl radical/carboxylate redox couple has been reported between 1.5 V and 1.7 V compared with SHE (ref. ^23^) (1.95–2.05 V compared with O_2_/O_2_^−^), which explains ^1^O_2_ formation according to Extended Data Fig. 4a. Alkyl peroxides (ROOR) such as di-tert-butyl, dicumyl and di*-n*-butyl peroxide have been reported not to form ^1^O_2_ (ref. ^45^). This can be understood by ROOR only breaking down to RO^–^ and RO^•^ at low potentials (–1.31 V to –1.15 V compared with SHE, that is, –0.86 V to –0.70 V compared with O_2_/O_2_^−^) (ref. ^47^). The RO^•^/RO^–^ couple (–0.06 V to 0.04 V compared with SHE, that is, 0.39–0.49 V compared with O_2_/O_2_^−^) could, if formed, oxidize O_2_^−^, but not to ^1^O_2_.

Proton-induced disproportionation that yields low but non-negligible ^1^O_2_ yields at a high pH of about 11 is consistent with reliable theoretical and experimental works. Using a high-level ab initio method, a previous study^17^ found that the reaction HO_2_ + O_2_^−^ → O_2_ + HO_2_ can proceed by both the singlet and triplet pathways, with the singlet pathway being only about 0.3 eV endoergic. Equivalent experiments exposing KO_2_ to H_2_O_2_ in toluene found about 0.2% ^1^O_2_ production, as measured using a chemical trap^14^. In this experiment, H_2_O_2_ was the proton source to first form HO_2_ (H_2_O_2_ + O_2_^−^ → HO_2_^−^ + HO_2_). The conditions in these theoretical and experimental studies correspond to a pH of about 11 (p*K*_a_ = 11.7 for H_2_O_2_), in which the reaction to ^1^O_2_ is only weakly driven. Conversely, ^1^O_2_ was not detected with alkyl hydroperoxides ROOH as the proton source (p*K*_a_ ≈ 12.6) (ref. ^45,48^), a result that insufficient driving forces can now explain.

### Relation between ${{\boldsymbol{E}}}_{{\genfrac{}{}{0ex}{}{1}{}{\bf{O}}}_{2}/{{\bf{O}}}_{{\bf{2}}}^{-}}^{\circ }$ and the ^1^O_2_ fraction

The electrochemiluminescence literature refers to energy-sufficient processes to form the electronically excited species^23,28,49^. For example, consider the generic redox couples R/R^•–^ and M^•+^/M (note that these could be, for example, ^3^O_2_/O_2_^−^ and RM^ox^/RM^red^). The process R^•–^ + M^•+^ → ^1^R^*^ + M is considered energy-sufficient to form the excited species ^1^R^*^, if $({E}_{{{\rm{M}}}^{\cdot +}/{\rm{M}}}^{\circ }-{E}_{{\rm{R}}/{{\rm{R}}}^{\cdot -}}^{\circ })F\ge \,-\Delta {H}^{\circ }(\,\genfrac{}{}{0ex}{}{1}{}\,{{\rm{R}}}^{* }\leftarrow {\rm{R}})$. This condition is fulfilled if the potential of the oxidizing redox couple exceeds the redox potential of the excited species: ${E}_{{{\rm{M}}}^{\cdot +}/{\rm{M}}}^{\circ }\ge {E}_{\genfrac{}{}{0ex}{}{1}{}{{\rm{R}}}^{* }/{{\rm{R}}}^{\cdot -}}^{\circ }={E}_{{{\rm{R}}/{\rm{R}}}^{\cdot -}}^{\circ }+\Delta {H}^{\circ }(\,\genfrac{}{}{0ex}{}{1}{}\,{{\rm{R}}}^{* }\leftarrow {\rm{R}})/F$. The connotation of energy-sufficient processes led to the interpretation that ${E}_{\genfrac{}{}{0ex}{}{1}{}{{\rm{R}}}^{* }/{{\rm{R}}}^{\cdot -}}^{\circ }$ or ${E}_{{\genfrac{}{}{0ex}{}{1}{}{\rm{O}}}_{2}/{{\rm{O}}}_{2}^{-}}^{\circ }$ establishes a threshold above which the excited species rather than ground state species forms^18,24,26^. Extended Data Fig. 4a shows that reaching this threshold potential (or the driving force for which this is exceeded) gives no indication about the extent to which ^1^O_2_ rather than ^3^O_2_ forms. An onset of ^1^O_2_ may be expected at ${\Delta G}_{1\leftarrow 3}^{\circ }$, but its formation will become significant only for driving forces –Δ*G*° > *λ*_3_, for which ^3^O_2_ formation slows down to benefit ^1^O_2_ formation.

### Thermodynamics in mixed alkali metal/TBA^+^ electrolytes

Superoxide disproportionation in Li^+^ and Na^+^ containing glyme electrolytes was found to always yield some ^1^O_2_ according to 2MO_2_ → M_2_O_2_ + *x*^3^O_2_ + (1 − *x*)^1^O_2_ (refs. ^13,16,18,19^). Li^+^ yielded small fractions (about 2%) at large kinetics, and Na^+^ yielded larger fractions (around 12%) at slow kinetics^16^. Often, electrolytes for non-aqueous metal-O_2_ batteries contain weakly Lewis acidic cations, such as tetrabutylammonium (TBA^+^) or other cations from ionic liquid electrolytes. In mixed Li^+^/TBA^+^ and Na^+^/TBA^+^ (1/1) electrolytes, the ^1^O_2_ yields increased to about 20 and 18%, respectively. The reasons for this behaviour must, hence, lie in (1) already sufficient driving forces for ^1^O_2_ formation in pure Li^+^ and Na^+^ electrolytes; and (2) increasing driving forces on adding TBA^+^.

M_2_O_2_ (M = Li^+^, Na^+^) are insoluble^50,51^ and the potential ${E}_{{\genfrac{}{}{0ex}{}{3}{}{\rm{O}}}_{2}/{{\rm{M}}}_{2}{{\rm{O}}}_{2}({\rm{s}})}^{\circ }\,=$ ${\Delta }_{{\rm{f}}}{G}^{\circ }({{\rm{M}}}_{2}{{\rm{O}}}_{2(\text{s})})/F=2.96\,{\rm{V}}$ compared with M^+^/M, therefore, fixed to the value obtained using the formation energy –Δ_f_*G*° of solid Li_2_O_2(s)_. Given that the superoxide/M_2_O_2(s)_ couple acts as the oxidant during superoxide disproportionation, the driving force is given by $-\Delta G=({E}_{{\rm{superoxide}}/{{\rm{M}}}_{2}{{\rm{O}}}_{2(\text{s})}}-{E}_{{\genfrac{}{}{0ex}{}{3}{}{\rm{O}}}_{2}/{\rm{superoxide}}})F$. Note that here superoxide does not denote a particular species, but it could be anything, including solid MO_2(s)_, solvated ${({{\rm{M}}}^{+}{{\rm{O}}}_{2}^{-})}_{n\ge 1,({\rm{sln}})}$ clusters and ion pairs, or the weakly coordinated ${({{\rm{TBA}}}^{+}{{\rm{O}}}_{2}^{-})}_{({\rm{sln}})}$. ${E}_{{\rm{superoxide}}/{\text{M}}_{2}{\text{O}}_{2(\text{s})}}$ cannot be directly measured but can be inferred from ${E}_{{\genfrac{}{}{0ex}{}{3}{}{\rm{O}}}_{2}/{{\rm{M}}}_{2}{{\rm{O}}}_{2(\text{s})}}$ and ${E}_{{\genfrac{}{}{0ex}{}{3}{}{\rm{O}}}_{2}/{\rm{superoxide}}}$. Using Δ*G* = –*zFE*, where *z* is the number of transferred electrons, it can be derived that ${E}_{{\rm{superoxide}}/{{\rm{M}}}_{2}{{\rm{O}}}_{2(\text{s})}}=2{E}_{{\genfrac{}{}{0ex}{}{3}{}{\rm{O}}}_{2}/{{\rm{M}}}_{2}{{\rm{O}}}_{2(\text{s})}}-{E}_{{\genfrac{}{}{0ex}{}{3}{}{\rm{O}}}_{2}/{\rm{superoxide}}}$. For the stable solid compounds (Li_2_O_2_, Na_2_O_2_, NaO_2_, K_2_O_2_ and KO_2_), tabulated formation energies Δ_f_*G*° can be found and the ${E}_{{\genfrac{}{}{0ex}{}{3}{}{\rm{O}}}_{2}/{{\rm{M}}}_{2}{{\rm{O}}}_{2}({\rm{s}})}^{\circ }$ and ${E}_{{\genfrac{}{}{0ex}{}{3}{}{\rm{O}}}_{2}/{\rm{M}}{{\rm{O}}}_{2}({\rm{s}})}^{\circ }$ be calculated as shown in Extended Data Fig. 5a. On the basis of this, KO_2_ is not expected to disproportionate to K_2_O_2_ and the K^+^-case, hence, not be further considered.

${E}_{{\genfrac{}{}{0ex}{}{3}{}{\rm{O}}}_{2}/{\rm{superoxide}}}$ require further consideration given the electrolyte-dependent solubilities of superoxide. Theoretical work shows that solvated ${({{\rm{Li}}}^{+}{{\rm{O}}}_{2}^{-})}_{n\ge 1,({\rm{sln}})}$ species are less stable in terms of Gibbs free energy than the bulk solid LiO_2(s)_, but as the cluster size *n* grows, the structure approaches bulk MO_2(s)_ and free energy approaches a constant value^16,50,51^. Aggregation into ${({{\rm{Li}}}^{+}{{\rm{O}}}_{2}^{-})}_{n > 1,({\rm{sln}})}$ clusters stabilizes the solvated species relative to separated ${({{\rm{Li}}}^{+}{{\rm{O}}}_{2}^{-})}_{({\rm{sln}})}$ species^16,34^. The Gibbs free energy grows, therefore, in the order of increasing solvation: LiO_2(s)_ < (Li^+^O_2_^–^)_*n*>1,(sln)_ < (Li^+^O_2_^–^)_(sln)_. Accordingly, the potentials in pure M^+^ electrolyte are in the order and within the limits ${E}_{{\genfrac{}{}{0ex}{}{3}{}{\rm{O}}}_{2}/{{\rm{LiO}}}_{2(\text{s})}} > {E}_{{\genfrac{}{}{0ex}{}{3}{}{\rm{O}}}_{2}/{({{\rm{Li}}}^{+}{{\rm{O}}}_{2}^{-})}_{n > 1({\rm{sln}})}} > {E}_{{\genfrac{}{}{0ex}{}{3}{}{\rm{O}}}_{2}/{({{\rm{Li}}}^{+}{{\rm{O}}}_{2}^{-})}_{({\rm{sln}})}}$. If TBA^+^ is present, the even weaker association in ${({{\rm{TBA}}}^{+}{{\rm{O}}}_{2}^{-})}_{({\rm{sln}})}$ extends the lower potential limit to ${E}_{{\genfrac{}{}{0ex}{}{3}{}{\rm{O}}}_{2}/{({{\rm{TBA}}}^{+}{{\rm{O}}}_{2}^{-})}_{({\rm{sln}})}}$. The values for these potential limits, as shown in Fig. 3a, Extended Data Fig. 5c,d, were estimated from cyclic voltammograms with the salt shifting from pure M^+^ to pure TBA^+^ (Extended Data Fig. 5b). The largely solvation-independent DMFc/DMFc^+^ redox couple was used as internal standard. Δ_f_*G*° of solid LiO_2(s)_ is not available, but ${E}_{{\genfrac{}{}{0ex}{}{3}{}{\rm{O}}}_{2}/{\rm{Li}}{{\rm{O}}}_{2}({\rm{s}})}$ may be estimated from cyclic voltammograms in poorly solvating electrolytes, in which large ${({{\rm{Li}}}^{+}{{\rm{O}}}_{2}^{-})}_{n,({\rm{sln}})}$ clusters approach the thermodynamics of LiO_2(s)_. The shift in the onset of O_2_ reduction in Li^+^ compared with TBA^+^ electrolyte at slow scan rates was taken as the difference between ${E}_{{{}^{3}{\rm{O}}}_{2}/{{\rm{LiO}}}_{2(\text{s})}}$ and ${E}_{{\genfrac{}{}{0ex}{}{3}{}{\rm{O}}}_{2}/{({{\rm{TBA}}}^{+}{{\rm{O}}}_{2}^{-})}_{({\rm{sln}})}}.{E}_{{\genfrac{}{}{0ex}{}{3}{}{\rm{O}}}_{2}/{({{\rm{Li}}}^{+}{{\rm{O}}}_{2}^{-})}_{({\rm{sln}})}}$ as the lower limit of potentials in pure Li^+^ electrolyte was estimated from the potential shift between pure Li^+^ and pure TBA^+^ electrolytes with the highly solvating solvent 1-methylimidazole. These were taken from ref. ^32^ and show a shift of 33 mV.

Extended Data Fig. 5d shows the analogous thermodynamics for Na^+^/TBA^+^ mixtures as shown in Fig. 3 for Li^+^/TBA^+^. Considerations as above for the relative stabilities of the Li superoxides apply analogously to the relative stability of Na superoxide clusters compared with the NaO_2(s)_ bulk. Theoretical work has similarly shown the stabilization by forming ${({{\rm{Na}}}^{+}{{{\rm{O}}}_{2}}^{-})}_{n > 1,({\rm{sln}})}$ clusters^52,53^. Extended Data Fig. 5e shows the NIR emission on adding KO_2_ into glyme electrolyte containing various Na^+^/TBA^+^ ratios. The data show that, even in a pure Na^+^ electrolyte, the driving force is sufficient for ^1^O_2_ formation and that added TBA^+^ increases the driving force and formation.

More generally, the electrolyte properties (solvent(s), salts(s) and their concentrations) will affect the reorganization energy and hence the maxima and crossing point of the two kinetic parabolas. The classical approach to accounting for this is the equation given by Marcus^54^, which connects the reorganization energy with the effective dielectric properties of the electrolyte and the separation of the redox centres. A lower dielectric constant and smaller separation will result in a larger reorganization energy. A refined equation by Marcus^55^ further takes into account the ionic environment. These considerations apply well, for example, to aqueous anionic redox couples and the series of alkali metal cations from Li^+^ to Cs^+^ as spectator cations, in which *λ* decreases^56^. However, caution is required with non-aqueous, low dielectric constant media, in which strong ion pairing occurs. Some works suggest an inverse trend for *λ* among the alkali metals^19,57^. Ion pairing and even clustering is particularly severe for (su)peroxide as the redox anions as discussed above. Superoxide forms in non-aqueous Li^+^ and Na^+^ electrolytes clusters^32,33^ and the peroxides are practically insoluble^51^. The order and extent to which the reorganization energy changes for superoxide oxidation in non-aqueous media among the alkali cations may, therefore, not be predicted straightforwardly and would merit further investigation. As we observe ^1^O_2_ at low driving force for the Na^+^ electrolyte, the reorganization energy appears sufficiently low therein.

### Wider relevance for life sciences and energy

Our study contributes to understanding how the pH affects the link between the four important reactive oxygen species (ROS) superoxide, peroxide, ^3^O_2_ and ^1^O_2_. Disproportionation is notably the pathway to maintain a low superoxide concentration. However, detoxification from superoxide produces the harmful ^1^O_2_. Superoxide occurs in cells in several organelles with different pH levels between 4.7 and 8, but the superoxide-degrading enzyme superoxide dismutase occurs only in neutral to basic organelles^35^. In pathological situations, the pH balance is known to be affected (typically towards lower pH) and therefore signalling, redox regulation and defence^58,59^. Our study contributes to the understanding of how the redox chemistry of superoxide, pH and ^1^O_2_ formation are linked. We noted that in non-aqueous media, superoxide in contact with CO_2_ forms ^1^O_2_. Given the ubiquity of CO_2_ in organelles containing superoxide, further investigations into the aqueous chemistry of CO_2_ and superoxide are warranted.

For energy applications, further relevance and future research directions emerge: (1) For suppressing ^1^O_2_, generally, the driving force should be decreased, and the reorganization energy for the superoxide oxidation reaction should be increased. The classical equation by Marcus^54^, which connects the reorganization energy with the effective dielectric properties of the electrolyte and the separation of the redox centres, applies well to aqueous anionic redox couples^56^. For non-aqueous, low dielectric constant media, in which strong ion pairing occurs, particularly so with superoxide, the change in reorganization energy among different cations and electrolytes may not be predicted straightforwardly and would merit further investigation. (2) Oxygen evolution catalysis from water: metal-superoxo species have been identified as preceding the O_2_ evolution on, for example, the extensively studied Ni(Fe)OOH or CoOOH catalysts^10^. The metal M^*n*–1/*n*^ redox couple is considered to oxidize the superoxo moiety to O_2_ (refs. ^10,60^). Some of the most active M^*n*–1/*n*^ metal redox couples are typically a few 100 mV above the ^1^O_2_/superoxide potential shown in Fig. 4a and provide, in principle, enough driving force for ^1^O_2_ evolution. For example, at pH 14 ${E}_{{\genfrac{}{}{0ex}{}{1}{}{\rm{O}}}_{2}/{{{\rm{O}}}_{2}}^{-}}^{\circ }=1.32\,{\rm{V}}$, ${E}_{{{\rm{Co}}}^{{\rm{II}}/{\rm{III}}}}^{\circ }\approx 1.25\,{\rm{V}}$, ${E}_{{{\rm{Co}}}^{{\rm{III}}/{\rm{IV}}}}^{\circ }\approx 1.5\,{\rm{V}}$, ${E}_{{{\rm{Ni}}}^{{\rm{II}}/{\rm{III}}}}^{\circ }\approx 1.52\,{\rm{V}}$ and ${E}_{{{\rm{Ni}}}^{{\rm{III}}/{\rm{IV}}}}^{\circ }\approx 1.6\,{\rm{V}}$ on the RHE scale (refs. ^10,60^). Further investigations specifically on ^1^O_2_ evolution in oxygen evolution catalysis are therefore warranted. (3) Both Li-stoichiometric^6^ and Li-rich transition metal (TM, for example, Ni, Mn and Co) oxide^2,7,61,62^ intercalation materials used for positive electrodes in Li- or Na-ion batteries are known to undergo parasitic lattice oxygen loss at high states of charge. Both the intercalation material and the electrolyte degrade, hampering long-term cyclability. However, non-matching patterns of O_2_ and CO_2_/CO release from electrolyte decomposition (all containing lattice O as shown by isotopic labelling^7,62^) and enhanced lattice O loss with surface carbonates present^7^ remain unexplained and highlight the need for a deeper understanding of the prevailing ROS and decomposition pathways. For LiNiO_2_, ^1^O_2_ evolution has been suggested to result from the disproportionation of oxide radicals^6^. More generally, ^1^O_2_ may evolve from superoxo species (at the lattice surface, in (su)peroxocarbonates^4,30^) at the available driving forces. The oxidants could be a combination of (su)peroxides (for example, coordinated by TMs or carbonates) that get stabilized by further reduction and TM redox, such as Co^III/IV^ or Ni^III/IV^. Further investigations into the involvement of ^1^O_2_ evolution in TM oxide outgassing and surface reactions are therefore warranted.

The results expand the current knowledge on the electrogeneration of excited species more generally and pose open questions about the origin. Specifically, the process is more effective than typically assumed, given that ${\Delta G}_{1\leftarrow 3}^{\circ } < {\Delta H}_{1\leftarrow 3}^{\circ }$ and that *Z*_el,1_ ≫ *Z*_el,3_. Electrogeneration of excited ROS has significance ranging from biological systems to energy storage. Reactive excited species in life are very broadly associated with pathogenic events^1,63^. Recombination reactions in redox flow batteries are recognized to cause self-discharge, but this has so far not been recognized to potentially form extremely energetic excited species. Soluble parasitic oxidized and reduced species at the cathode and anode of Li- and Na-ion batteries may recombine to form energetic excited species.

## Online content

Any methods, additional references, Nature Portfolio reporting summaries, source data, extended data, supplementary information, acknowledgements, peer review information; details of author contributions and competing interests; and statements of data and code availability are available at 10.1038/s41586-025-09587-7.

## Supplementary information

Peer Review File

## Source data

Source Data Fig. 1

Source Data Fig. 2

Source Data Fig. 3

Source Data Fig. 4

## Data Availability

The data that support the plots in this paper and other findings of this study are available from the corresponding author upon reasonable request. Source data are provided with this paper.

## Code availability

Figures were made using MATLAB and the built-in toolboxes. Simulations in Fig. 2 were done following the model and equations given in the Methods. The scripts used in this study are available from the corresponding author upon reasonable request.
